# Innate sensing of picornavirus infection involves cGAS-STING-mediated antiviral responses triggered by mitochondrial DNA release

**DOI:** 10.1371/journal.ppat.1011132

**Published:** 2023-02-06

**Authors:** Huisheng Liu, Zixiang Zhu, Qiao Xue, Fan Yang, Zongqiang Li, Zhaoning Xue, Weijun Cao, Jijun He, Jianhong Guo, Xiangtao Liu, Andrew E. Shaw, Donald P. King, Haixue Zheng

**Affiliations:** 1 State Key Laboratory of Veterinary Etiological Biology; College of Veterinary Medicine, Lanzhou University, WOAH/National reference laboratory for foot-and-mouth disease; Lanzhou Veterinary Research Institute, Chinese Academy of Agricultural Sciences, Lanzhou, China; 2 State Key Laboratory of Membrane Biology, Beijing Advanced Innovation Center for Structural Biology, School of Life Sciences, Tsinghua University, Beijing, China; 3 The Pirbright Institute, Pirbright, Surrey, United Kingdom; University of California, Irvine, UNITED STATES

## Abstract

Cyclic GMP-AMP synthase (cGAS) plays a key role in the innate immune responses to both DNA and RNA virus infection. Here, we found that enterovirus 71 (EV-A71), Seneca Valley virus (SVV), and foot-and-mouth disease virus (FMDV) infection triggered mitochondria damage and mitochondrial DNA (mtDNA) release *in vitro and vivo*. These responses were mediated by picornavirus 2B proteins which induced mtDNA release during viral replication. SVV infection caused the opening of mitochondrial permeability transition pore (mPTP) and led to voltage-dependent anion channel 1 (VDAC1)- and BCL2 antagonist/killer 1 (Bak) and Bak/BCL2-associated X (Bax)-dependent mtDNA leakage into the cytoplasm, while EV-A71 and FMDV infection induced mPTP opening and resulted in VDAC1-dependent mtDNA release. The released mtDNA bound to cGAS and activated cGAS-mediated antiviral immune response. cGAS was essential for inhibiting EV-A71, SVV, and FMDV replication by regulation of IFN-β production. cGAS deficiency contributed to higher mortality of EV-A71- or FMDV-infected mice. In addition, we found that SVV 2C protein was responsible for decreasing cGAS expression through the autophagy pathway. The 9th and 153rd amino acid sites in 2C were critical for induction of cGAS degradation. Furthermore, we also show that EV-A71, CA16, and EMCV 2C antagonize the cGAS-stimulator of interferon genes (STING) pathway through interaction with STING, and highly conserved amino acids Y155 and S156 were critical for this inhibitory effect. In conclusion, these data reveal novel mechanisms of picornaviruses to block the antiviral effect mediated by the cGAS-STING signaling pathway, which will provide insights for developing antiviral strategies against picornaviruses.

## Author summary

It is widely known that picornaviruses inhibit host innate immune responses by regulating retinoic acid-inducible gene I (RIG-I)-like receptors (RLRs) pathways. However, the impact of picornavirus infection on cytosolic DNA sensors remains unknown. Here, we found that picornavirus infection causes mitochondrial dysfunction that triggers mitochondrial DNA release via mitochondrial permeability transition pore (mPTP). The released mitochondrial DNA subsequently activates cyclic GMP-AMP synthase-stimulator of interferon genes (cGAS-STING)-mediated signaling transduction, contributing to type I interferon expression. In addition, we identified novel antagonistic mechanisms by which picornavirus 2C proteins antagonize cGAS-STING signaling pathway activation and determined new antagonistic sites in 2C proteins. We conclude that the cGAS-STING signaling pathway is critical for suppressing picornavirus infections, and picornaviral 2C proteins block its activation through multiple strategies to favor virus replication. For the first time, our study determines the antiviral role of cGAS-STING signaling pathway in picornavirus infection and describes a picornavirus-mediated inhibitory effect on DNA-triggered innate immune signaling.

## Introduction

The family of *Picornaviridae* consists of a variety of RNA viruses, including poliovirus, enterovirus 71 (EV-A71), Seneca Valley virus (SVV), encephalomyocarditis virus (EMCV), and foot-and-mouth disease virus (FMDV). Many are important human and livestock pathogens, affecting the hand, foot and mouth, central nervous system, liver, heart, and respiratory and gastrointestinal tracts. All members of the *Picornaviridae* family are nonenveloped and positive single-stranded RNA viruses encoding a single polyprotein that is post-translationally cleaved into mature structural and non-structural proteins. Some enterovirus genomes also harbour an upstream open reading frame (uORF) [1,2].

The innate immune system is the first line of the host’s defense that protects against invading pathogens. Host pattern recognition receptors (PRRs) such as RIG-like receptors (RIG-I, MDA5, and LGP2), NOD-like receptors (NOD2), cyclic GMP-AMP synthase (cGAS), and interferon gamma inducible protein 16 (IFI16) play a critical role in innate immunity where they interact with pathogen-associated molecular patterns (PAMPs) to induce interferon (IFN) production which exert antiviral functions [3]. cGAS, as a key PRR, is activated by viral or cytosolic DNA [4] to bind to the stimulator of interferon genes (STING) which further activates TANK-binding kinase 1 (TBK1) and interferon regulatory factor 3 (IRF3) to induce IFNs production [5]. STING also activates autophagy (a fundamental role in cellular, tissue, and organismal homeostasis and is regulated by autophagy-related (ATG) genes) through a mechanism that is independent of TBK1 activation [6].

To counteract host antiviral responses and maintain viral replication, various DNA viruses have evolved mechanisms to antagonize the activation of the cGAS-STING signaling pathway [7,8]. cGAS is at the intersection of DNA and RNA virus-sensing networks [9–12]. The relationship between RNA viruses and cGAS has also been reported recently. RNA virus infection can trigger the mitochondrial DNA (mtDNA) leakage into the cytoplasm using BCL2 antagonist/killer 1 (Bak) and Bak/BCL2-associated X (Bax), resulting in the activation of cGAS-STING signaling pathway [13]. These effects can be inhibited via the degradation of cGAS or STING [14,15]. For example, influenza A virus (IAV) activates mtDNA-mediated antiviral immune responses [16]. Dengue virus (DENV) NS2B degrades cGAS to prevent mtDNA sensing by cGAS during viral infection [14,17]. Zika virus (ZIKV) evades antiviral response by inducing the cleavage of cGAS via caspase-1, and cleavage of STING by viral NS2B3 protease [15,18]. Some cellular proteins, such as interleukin-1β and TDP-43, induce mtDNA release to activate innate immune signaling via cGAS-STING [19,20]. TDP-43 can mislocalize into mitochondria, opening the mitochondrial permeability transition pore (mPTP) and resulting in voltage-dependent anion channel 1 (VDAC1)-dependent mtDNA leakage into the cytoplasm [19].

Although the pathogenic mechanisms by which viruses causes diseases have been explored, the pathogenic mechanisms underlying picornavirus-induced immune evasion are not fully understood. Work to understand the inhibition of innate immune responses by picornaviruses has focused on retinoic acid-inducible gene I (RIG-I)-like receptors (RLRs) and nucleotide-binding domain and leucine-rich repeat-containing receptors (NLRs) pathways. SVV 2C and 3C proteins suppress the host type I IFN production by cleaving or degrading innate immune signaling molecules in the RLRs pathways [21,22]. FMDV 2B or 2C antagonizes NOD2-, RIG-I-, MDA5-, or LGP2-mediated innate immune response [23,24], and FMDV L^pro^ and 3C^pro^ inhibits host innate immune response to promote viral replication in different manners [25]. Furthermore, EV-A71 3C inhibits RIG-I-mediated IRF3 activation [26] and the 2C protein inhibits type I IFN production and NF-κB activation by suppressing IκB kinase phosphorylation or binding to RelA(p65) [27,28]. FMDV L^pro^ and EV-A71 2A proteins can induce cleavage of eukaryotic translation initiation factor 4 G (eIF4G), resulting in inhibition of host protein synthesis and the antiviral response [25,29]. FMDV 2BC protein is an intermediate precursor that is processed into 2B and 2C during viral infection and it inhibits protein trafficking between the endoplasmic reticulum (ER) and the Golgi apparatus [24]. Picornavirus 2B protein is also involved in cytopathic effects, cellular protein secretion, and apoptotic responses [24]. Picornavirus 2C protein is a highly conserved and complex non-structural protein, but its functions are not well understood. The 2C protein contains an N-terminal membrane-binding domain, a central ATPase domain, and a C-terminal helical domain harboring a loop. The specific domains confer cell membranes binding, membrane rearrangement, RNA binding, formation of the viral replication complex, or immune evasion, revealing the multiple roles of 2C protein of picornaviruses [30]. The impacts of picornavirus infection on the cGAS-STING signaling pathway remains unknown.

The aim of this study was to examine the mechanism by which picornavirus infection regulates the cGAS-STING signaling pathway. We found that SVV, FMDV, and EV-A71 infection induced mitochondrial injury and opened the mPTP, resulting in VDAC1- or Bax/Bak-dependent mtDNA release. The released mtDNA in the cytoplasm activates the cGAS-STING signaling pathway, resulting in inhibition of picornaviral replication. SVV 2C protein impaired cGAS-mediated antiviral response through degradation of cGAS protein via autophagy pathway, and EV-A71, CA16, and EMCV 2C antagonized the cGAS-STING signaling pathway through interaction with STING. Our findings demonstrate that cGAS-STING pathway plays a critical role to inhibit picornaviral infection and revealed novel strategies for these viruses to evade these mechanisms.

## Results

### Picornavirus induced mitochondrial damage

Mitochondria play an important role in critical central metabolic pathways and integrate into the intracellular signaling networks that regulate diverse cellular functions [31]. However, the impact of picornavirus infection on mitochondrial function remains poorly understood. The JC-1 probe, Rhod-2 AM probe, mitoSOX red mitochondrial superoxide indicator, and calcein-AM staining solution are well-known reagents for detecting mitochondrial function [19,32,33]. Thus, these reagents were selected to monitor mitochondrial damage after EV-A71, SVV, or FMDV infection.

**Fig 1:**
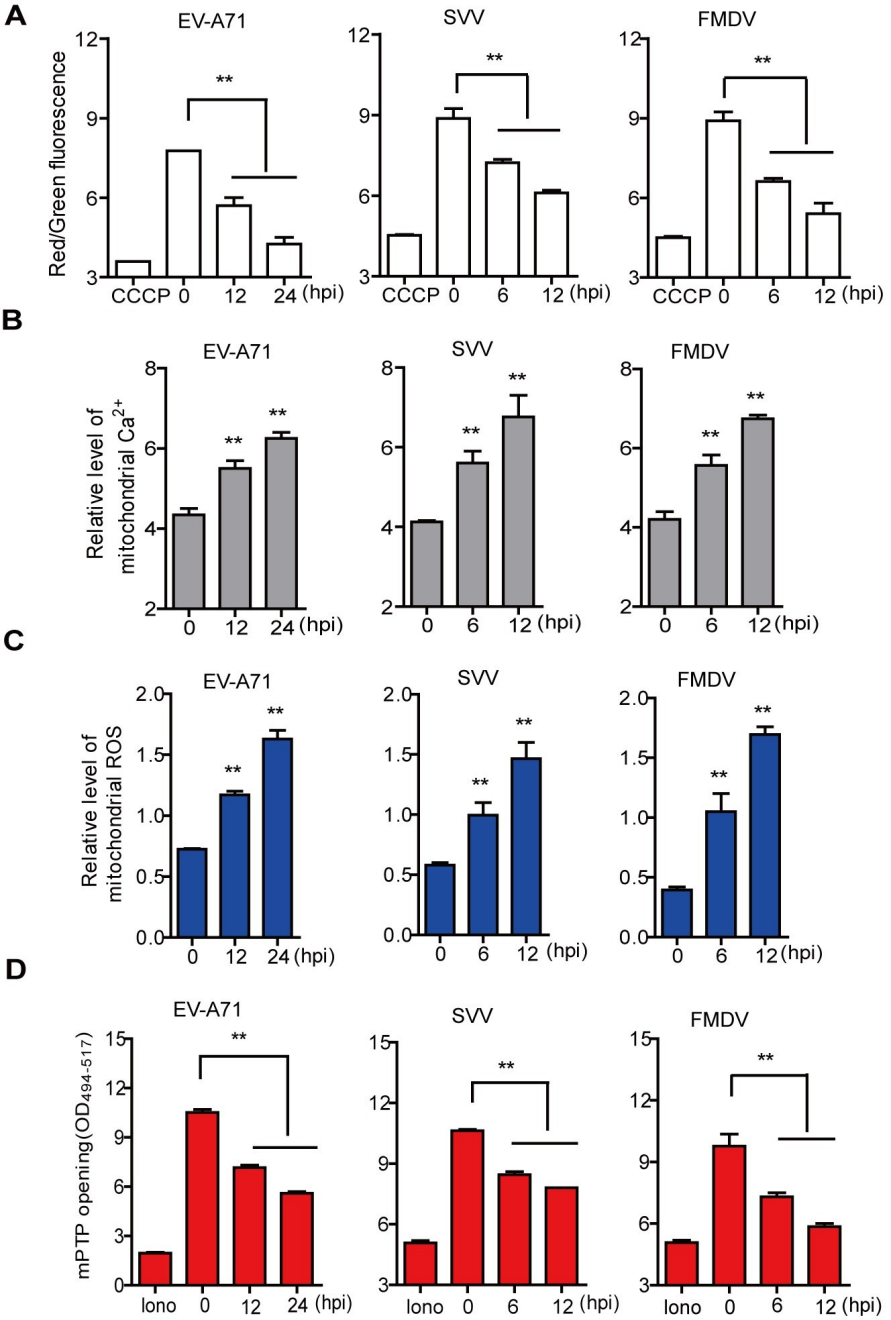
Mitochondrial damage induced by picornavirus infection. HeLa cells were mock-infected and infected with EV-A71 (MOI 1) for 0, 12, and 24 h. PK-15 cells were mock-infected and infected with SVV (MOI 1) or FMDV (MOI 0.5) for 0, 6, and 12 h. The ΔΨm (A), mitochondrial Ca^2+^ concentration (B), mitochondrial ROS (C), and mPTP opening (D) were detected using JC-1, Rhod-2 AM, mitoSOX, and calcein-AM, respectively. Data for CCCP (A) and lono (D) positive controls are shown. Error bars show standard deviation. **, *P*<0.01.

In conditions of high mitochondrial membrane potential (ΔΨm), JC-1 aggregates in the mitochondria matrix and produces red fluorescence. In contrast, when ΔΨm is low, only monomeric JC-1 is present which produces green fluorescence and therefore the ratio of red/green fluorescence is indicative of ΔΨm. In these studies, carbonyl cyanide m-chlorophenyl hydrazone (CCCP) which inhibits mitochondrial oxidative phosphorylation was used as a positive control to decrease ΔΨm. Infection of cells with EV-A71, SVV, or FMDV significantly reduced the ratio of red/green fluorescence for JC-1, indicating that picornavirus infection disrupts ΔΨm (Figs 1A and S1A).

The concentration of mitochondrial free Ca^2+^ in picornavirus-infected cells was measured using Rhod-2 AM as an indicator of mitochondrial damage [34]. Rhod-2 AM is localized in mitochondria and is cleaved by lactonase to form Rhod-2, which binds to Ca^2+^ and produces fluorescence in proportion to the Ca^2+^ concentration in mitochondria. In comparison to mock-inefected cells, EV-A71, SVV, or FMDV significantly induced the accumulation of Ca^2+^ into mitochondria as infection progressed (Fig 1B).

Mitochondrial reactive oxygen species (ROS) can also be used to monitor mitochondrial damage and can be detected using the mitoSOX red mitochondrial superoxide indicator. This fluorogenic dye enters mitochondria and is oxidized by superoxide anions, resulting red fluorescence. The mitochondrial ROS in the EV-A71-, SVV-, or FMDV-infected cells was detected by mitoSOX, which showed that EV-A71, SVV, or FMDV infection significantly enhanced mitochondrial ROS (Fig 1C).

Mitochondrial permeability transition pore (mPTP) plays an important physiological role in maintaining mitochondria homeostasis. The state of mPTP can be evaluated by calcein-AM and CoCl_2_. Calcein-AM is hydrolyzed by esterase to form calcein, producing green fluorescence, which can be quenched by Co^2+^. In these experiments, a calcium ionophore lonomycin (lono) was selected as a positive control. Lono promotes mPTP opening and the combination of calcein and Co^2+^, which leads to the quenching of the fluorescence signal. Therefore, the fluorescence intensity of calcein negatively correlated with openness of mPTP. Like the lono, EV-A71, SVV, or FMDV infection also decreased the fluorescence intensity of calcein, indicating that EV-A71, SVV, or FMDV infection significantly induced mPTP opening (Figs 1D and S1B). Together, these results suggested that EV-A71, SVV, or FMDV infection decreased ΔΨm, enhanced the Ca^2+^ concentration and ROS in the mitochondria, and induced mPTP opening, resulting in mitochondrial damage and dysfunction.

### Picornavirus induced release of mitochondrial DNA (mtDNA)

**Fig 2:**
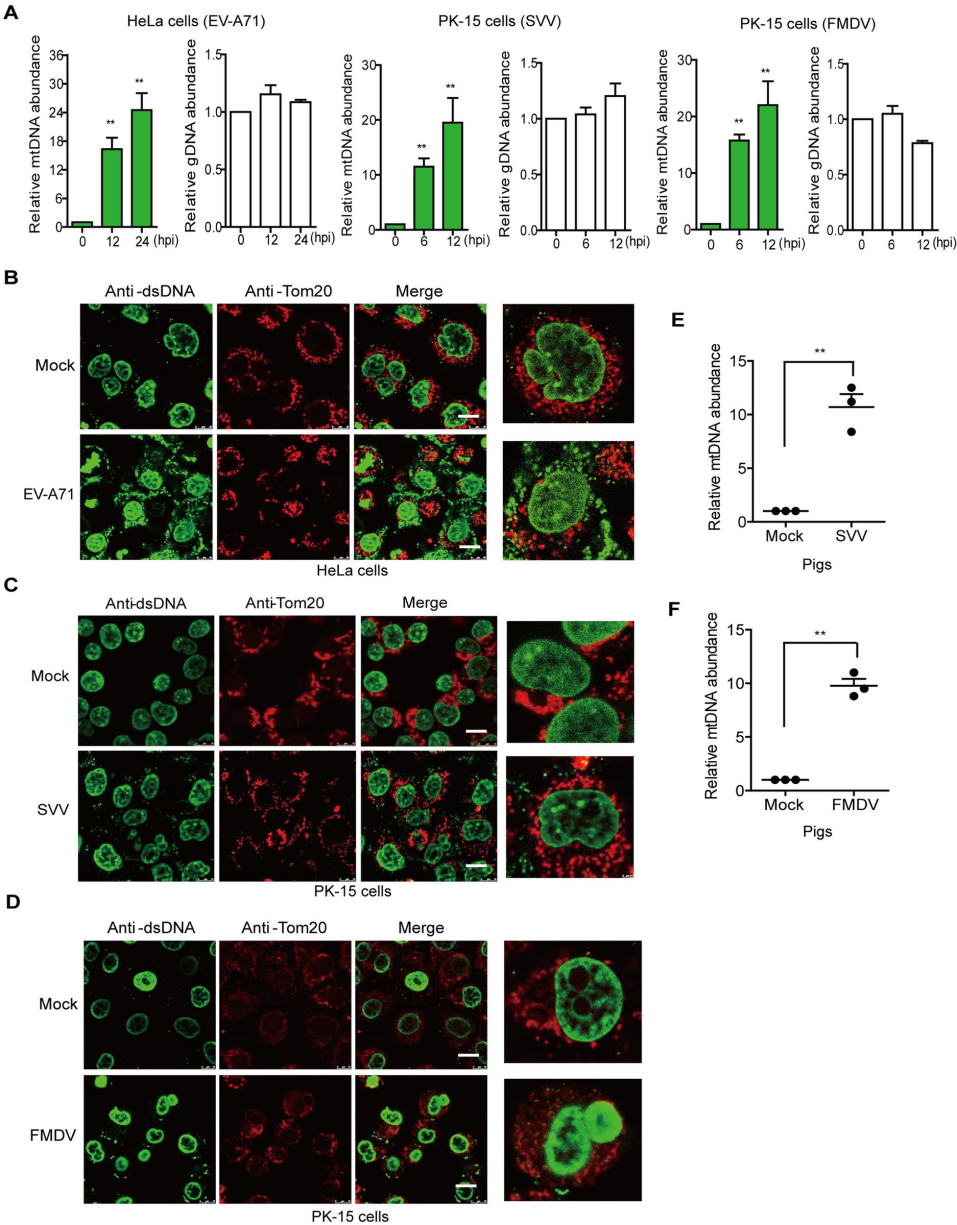
Picornavirus induced mtDNA release. HeLa cells were mock-infected or infected with EV-A71 (MOI 1) for 0, 12, and 24 h, while PK-15 cells were mock-infected or infected with SVV (MOI 1) or FMDV (MOI 0.5) for 0, 6, and 12 h. The lysates were immunoprecipitated with anti-cGAS antibody. The mtDNA and gDNA in cGAS pulldown samples were detected and analyzed by qPCR. The EGFP DNA was used as an internal control (A). HeLa cells were mock-infected or infected with EV-A71 (MOI 1) for 24 h. PK-15 cells were mock-infected or infected with SVV (MOI 1) or FMDV (MOI 0.5) for 12 h. The mtDNA release was evaluated by IFA. Cells were double-immunostained for detection of Tom20 (red) and dsDNA (green); cellular nuclei were counterstained with 4’,6-diamidino-2-phenylindole (DAPI) (blue) (B, C, and D). Scale bar, 10 μm. Oropharyngeal tonsils collected from pigs infected with SVV (E) or FMDV (F) were analyzed for mtDNA release. Error bars show standard deviation. **, *P*<0.01.

cGAS senses viral DNA and cellular DNA that is abnormally present within the cytosol to activate downstream signaling. Mitochondria damage or dysfunction leads to leakage of mtDNA into the cytosol, which can be recognized by cGAS. During this study, we evaluated mtDNA that was bound to cGAS during picornavirus infection. The cells infected with EV-A71, SVV, or FMDV were collected and the lysates were immunoprecipitated with anti-cGAS antibody. The quantitative PCR (qPCR) was used to assess the abundance of genomic DNA (gDNA) and mtDNA in the pulldown samples. Previous studies showed that the enhanced green fluorescent protein (EGFP) expression plasmid could be added to the pulldown samples to quantify the presence of gDNA and mtDNA [14]. Therefore, the EGFP expression plasmid was selected in the present study to quantify the presence of specific DNA fragments in the pulldown samples. The relative abundance of mtDNA in EV-A71-, SVV-, and FMDV-infected cells was significantly enhanced (>10 fold) compared to that in the mock-infected cells, which indicated that the mtDNA was leaked into the cytosol and bound to cGAS during picornavirus infection. In contrast, there was no significant difference in the abundance of gDNA bound to cGAS in the mock-infected and picornavirus-infected cells (Fig 2A). Similar results were also observed in EV-A71-infected HT-29 cells (S2A Fig).

mtDNA release was further detected by IFA using anti-dsDNA and anti-Tom20 (indicator of mitochondria) antibodies. The mtDNA in the mock-infected cells was mainly distributed in the mitochondria, whereas cytosolic mtDNA could be detected after EV-A71, SVV, or FMDV infection (Figs 2B, 2C and 2D and S2B). Altogether, our results confirmed that EV-A71, SVV, and FMDV infection induced mtDNA release.

Oropharyngeal tonsils are one of the critical replication sites for FMDV and SVV [35,36]. To assess whether mtDNA was released in FMDV- or SVV-infected pigs, oropharyngeal tonsils from SVV- [37] or FMDV-infected [38] pigs were collected at 3 d post-infection (dpi). FMDV and SVV RNA were detectable in the submandibular lymph nodes and tonsil tissues [37,38]. The mtDNA release state was evaluated by qPCR. SVV or FMDV infection considerably induced mtDNA release in pigs (Fig 2E and 2F).

cGAS senses DNA to produce 2’3’-cyclic GMP-AMP (2’3’-cGAMP) that binds and activates the adaptor protein STING, which triggers the innate immune response. The generation of 2’3’-cGAMP and STING phosphorylation at S366 are essential for activation of the cGAS-STING signaling pathway [39]. Therefore, 2’3’-cGAMP and the phosphorylation of STING in FMDV-, SVV-, or EV-A71-infected cells were further detected. Poly(dA:dT) was used as a positive control. FMDV, SVV, and EV-A71 infection induced increase of 2’3’-cGAMP and phosphorylation of STING (S3 Fig). Together, these results suggested that the release of mtDNA induced by FMDV, SVV, and EV-A71 infection activates the cGAS-STING signaling pathway.

### Picornavirus infection induced mtDNA release into the cytoplasm via mPTP

**Fig 3:**
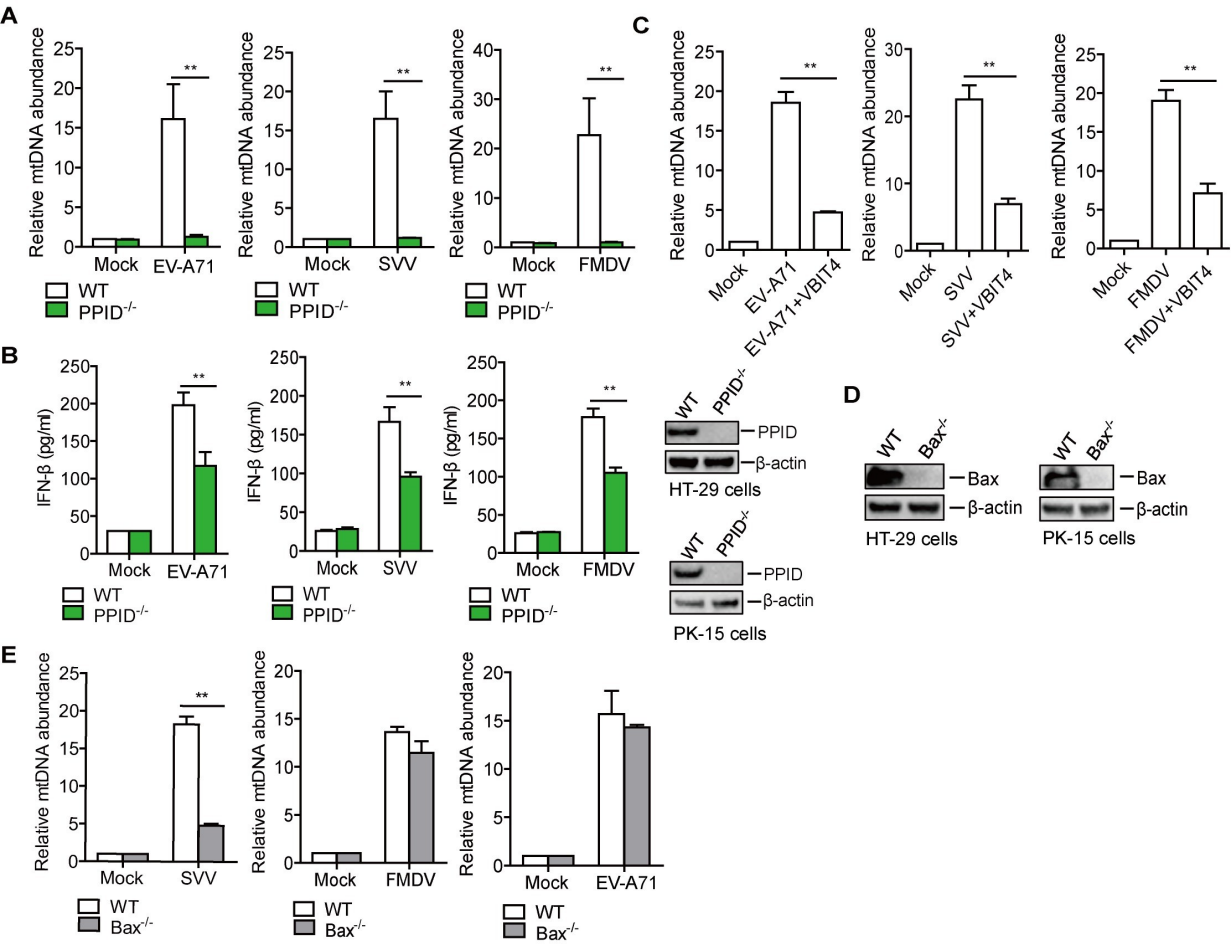
Picornavirus induced mtDNA release into the cytoplasm via mPTP. HT-29 WT and PPID^-/-^ cells were mock-infected or infected with EV-A71 (MOI 1) for 24 h, while PK-15 WT or PPID^-/-^ cells were mock-infected or infected with SVV (MOI 1) or FMDV (MOI 0.5) for 12 h. The mtDNA release was evaluated by qPCR (A). The expression of the IFN-β protein was detected by ELISA kit, and the expression of PPID was confirmed by Western blotting (B). (C) HT-29 cells were mock-infected or infected with EV-A71 (MOI 1), while PK-15 cells were mock-infected or infected with SVV (MOI 1) or FMDV (MOI 0.5). After 1 h of incubation of the virus or DMEM, cells were treated with DMSO or VBIT4 inhibitor (10 uM) for 24 or 12 h. The mtDNA release was then detected by qPCR. The expression of Bax protein in the WT and Bax^-/-^ cells was confirmed by western blotting (D) and these cells were used to measure mtDNA release after infection with EV-A71 (HT-29 cells at MOI 1) or SVV (PK-15 cells at MOI 1) or FMDV (PK-15 cells at MOI 0.5) for 12 h (E). Error bars show standard deviation. **, *P*<0.01.

After confirming that picornavirus infection induces mtDNA release, we next sought to identify the mechanism of mtDNA release into the cytosol. PPID (also called Cyclophilin D, a peptidyl-prolyl isomerase F), a component of mPTP, which plays important roles in mPTP opening and mtDNA release, was used to evaluate the role of mPTP during picornavirus infection [40]. To investigate whether mPTP was involved in picornavirus-induced mtDNA release, PPID knockout (PPID^-/-^) HT-29 and PK-15 cells were generated respectively, and the mtDNA release state in the PPID^-/-^ cells was evaluated. Our results showed that genetic deletion of PPID completely abolished EV-A71-, SVV-, and FMDV-mediated mtDNA release (Fig 3A) and the IFN-β production was considerably decreased in the picornavirus-infected PPID^-/-^ cells compared to that in the wildtype (WT) cells (Fig 3B). The knockout of PPID was confirmed by Western blotting (Fig 3B). In addition, the virus titers were significantly higher in the PPID^-/-^ cells than in the WT cells during viral infection (S4 Fig).

The voltage-dependent anion channel 1 (VDAC1) participates in mtDNA release under oxidative stress conditions [41]. BCL2 antagonist/killer 1 (Bak) and Bak/BCL2-associated X (Bax) regulate outer mitochondrial membrane permeability and induce mtDNA release into the cytoplasm in stress-induced apoptosis. mPTP is located at the inner mitochondrial membrane, while VDAC1 and Bak/Bax are located at the outer mitochondrial membrane [41,42]. Here, a VDAC1 oligomerization inhibitor VBIT-4 was used to evaluate the role of VDAC1 in picornavirus infection. Inhibition of VDAC1 oligomerization significantly decreased EV-A71-, SVV-, and FMDV-mediated mtDNA release (Fig 3C). The Bax gene was knocked out in HT-29 and PK-15 cells to abolish Bak/Bax-mediated outer mitochondrial membrane permeability (Fig 3D), and the mtDNA release induced by picornavirus infection was evaluated and compared in WT and Bax^-/-^ cells. The deletion of Bax significantly decreased SVV-mediated mtDNA release but had no effect on EV-A71- or FMDV-induced mtDNA release (Fig 3E). Our results indicated that SVV infection induced mPTP opening and led to VDAC1- and Bak/Bax-dependent mtDNA release. EV-A71 and FMDV infection induced mPTP opening, leading to VDAC1-dependent and Bak/Bax-independent mtDNA release.

### Picornavirus 2B proteins contributed to mtDNA release

**Fig 4:**
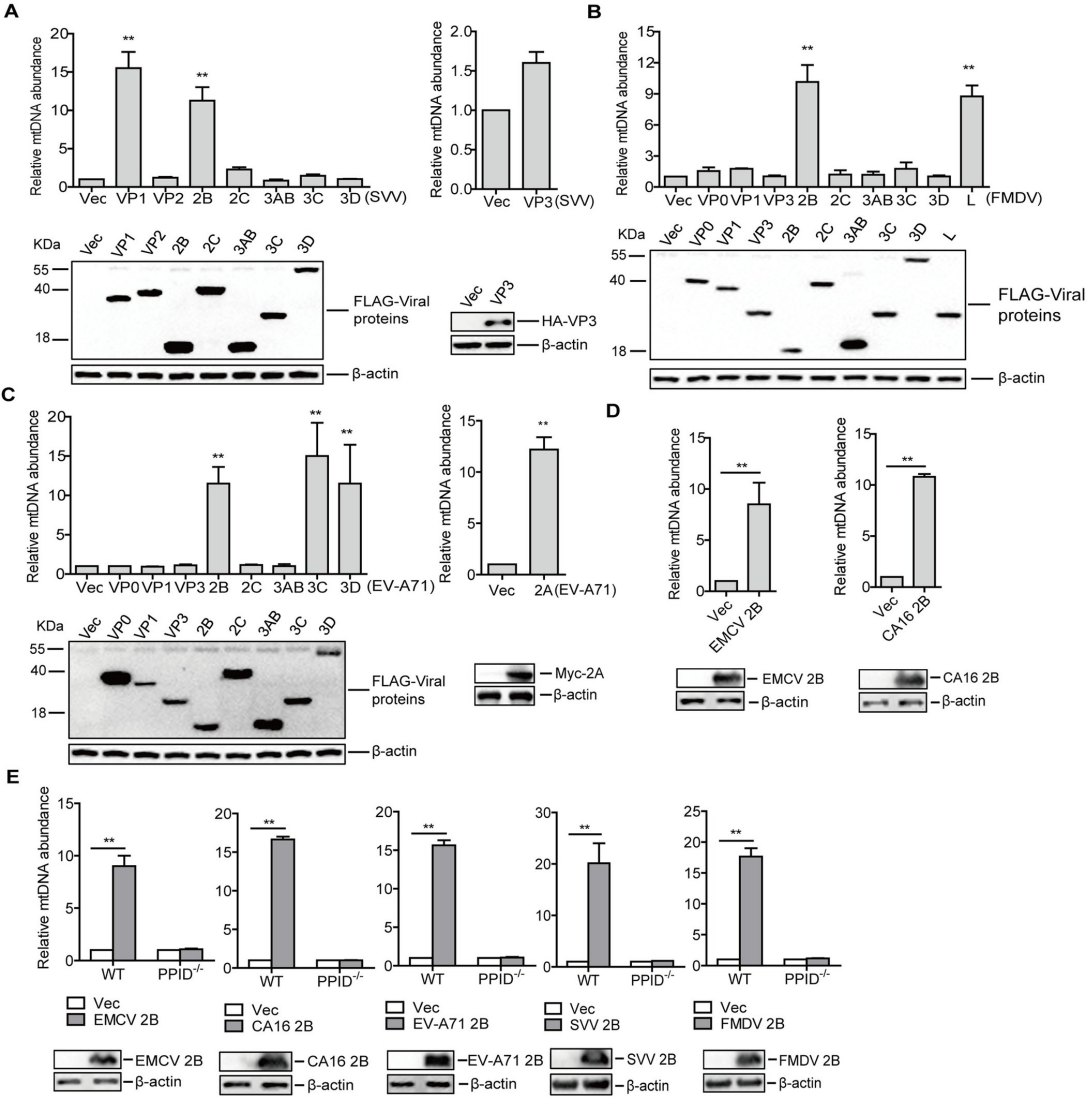
Picornavirus 2B proteins promoted mtDNA release. PK-15 cells were transfected with 2 μg of empty vector, the indicated SVV protein expressing plasmids (A) or FMDV protein expressing plasmids (B), and HeLa cells were transfected with 2 μg of empty vector or EV-A71 protein expressing plasmids (C). At 24 hpt, the mtDNA release was analyzed by qPCR. The expression of viral proteins was determined by Western blotting. (D) HeLa cells were transfected with 2 μg of empty vector, EMCV 2B or CA16 2B expressing plasmids for 24 h. The mtDNA release was detected by qPCR. (E) HT-29 WT and PPID^-/-^ cells were transfected with 2 μg of empty vector, EMCV 2B, CA16 2B, or EV-A71 2B expressing plasmids for 24 h, and PK-15 WT or PPID^-/-^ cells were transfected with 2 μg of empty vector, SVV 2B or FMDV 2B expressing plasmids for 24 h. The mtDNA release was detected by qPCR. Error bars show standard deviation. **, *P*<0.01.

To investigate the viral proteins responsible for the induction of mtDNA release during picornaviral infection, cells were transfected with the plasmids expressing various FLAG-tagged viral proteins. Our results showed that overexpression of SVV VP1 or 2B protein significantly caused mtDNA release in PK-15 cells (Fig 4A), overexpression of FMDV 2B or L^pro^ protein significantly induced mtDNA release in PK-15 cells (Fig 4B), and overexpression of EV-A71 2A, 2B, 3C^pro^, or 3D^pol^ protein significantly triggered mtDNA release in HeLa cells (Fig 4C).

The similar observed responses for the 2B proteins of EV-A71, FMDV and SVV implied that picornaviral 2B proteins might share a common mechanism to contribute to the induction of mtDNA release. To widen these findings beyond the three picornaviruses included this study, the impact of 2B proteins for other picornaviruses on mtDNA release was subsequently investigated, where results showed that encephalomyocarditis virus (EMCV) 2B- and coxsackievirus A16 (CA16) 2B-expressing plasmids also elevated mtDNA release (Fig 4D).

To further confirm the impact of picornavirus 2B proteins on mtDNA release, WT and PPID^-/-^ cells were transfected with empty vector, SVV 2B-, FMDV 2B-, EV-A71 2B-, EMCV 2B-, or CA16 2B-expressing plasmids, respectively. mtDNA release was determined by qPCR. All of these picornaviral 2B proteins induced a significant mtDNA release in the WT cells, while they did not trigger mtDNA release in the PPID^-/-^ cells (Fig 4E). Together, our results indicated that significant mtDNA release was triggered during picornaviruses infection, and the picornaviral 2B proteins were involved in this process through a common mechanism involving mPTP.

### Effect of cGAS deficiency on picornaviruses replication *in vitro*

**Fig 5:**
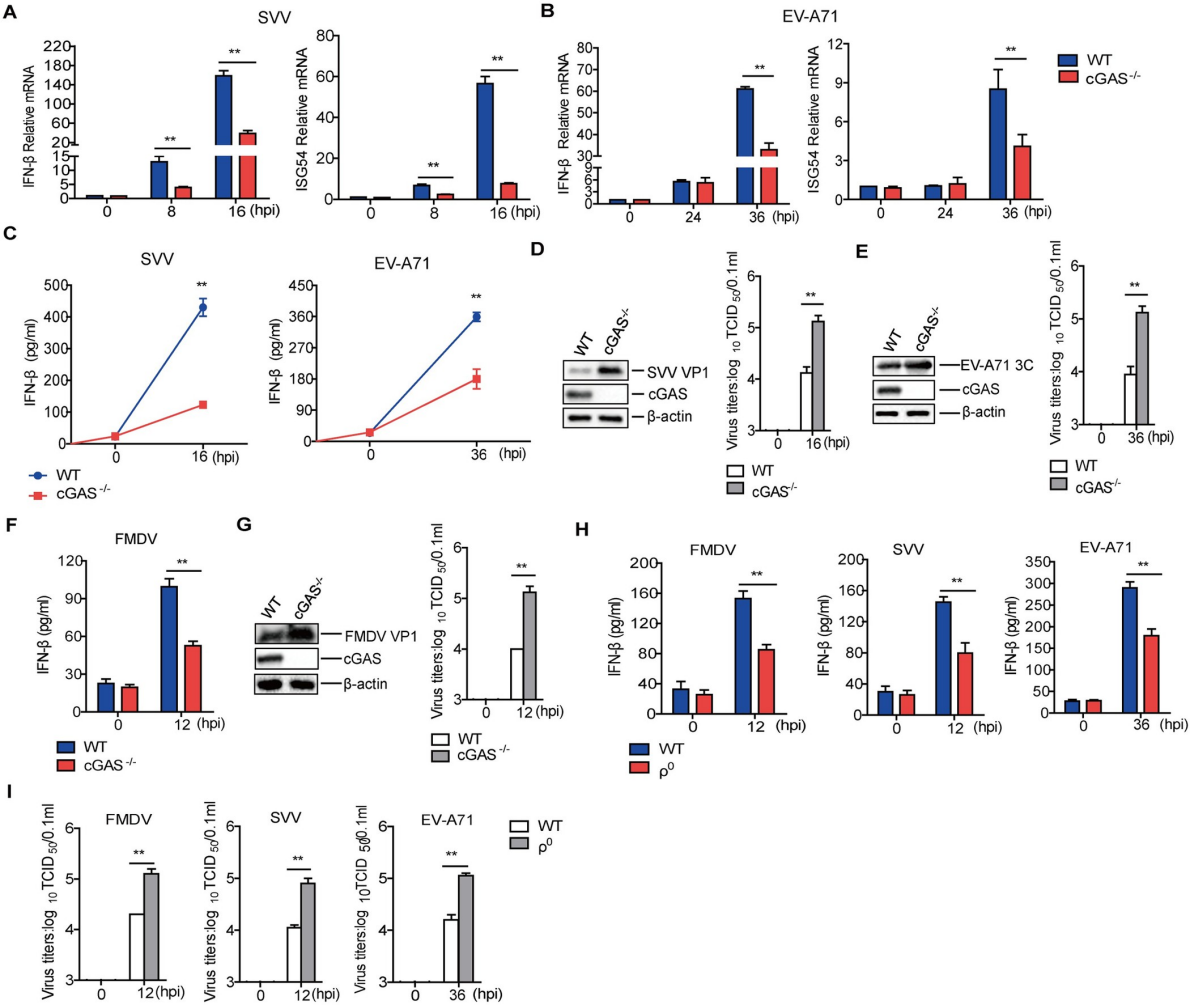
Effect of cGAS deficiency on picornaviruses replication *in vitro*. IFN-β and ISG54 mRNA expression in cGAS^-/-^ HeLa cells infected with SVV (A), and cGAS^-/-^ HT-29 cells infected with EV-A71 (B); IFN-β protein expression is also shown (C). Western blotting was used to detect the expression of SVV VP1 protein (D) and EV-A71 3C protein (E) and viral titers were determined by TCID_50_ assay. Bone marrow-derived macrophages from the WT or cGAS^-/-^ mice were infected with FMDV and IFN-β protein in the supernatant was determined by ELISA (F). The expression of FMDV VP1 protein was detected by Western blotting, and the viral titers were determined by TCID_50_ assay (G). WT and ρ^0^ PK-15 cells were infected with SVV or FMDV and WT and ρ^0^ HT-29 cells were infected with EV-A71. IFN-β protein expression level was determined by ELISA (H), and the viral titers were determined by TCID_50_ assay (I). Error bars show standard deviation. **, *P*<0.01.

mtDNA is recognized by the DNA sensor cGAS. The binding of mtDNA to cGAS activates the cGAS-STING signaling pathway and activates innate immune responses. Thus, we further investigated the consequence of picornavirus-triggered mtDNA release in the induction of cGAS-dependent IFN-β expression and antiviral effect. IFN-β and ISG54 mRNA expression (Fig 5A and 5B), as well as the IFN-β protein expression (Fig 5C) were significantly decreased in cGAS^-/-^ cells compared to WT cells during SVV or EV-A71 infection. The virus yields in SVV- or EV-A71-infected cGAS^-/-^ cells were subsequently determined. The virus titers were remarkably higher in the cGAS^-/-^ cells compared to that in the WT cells (Fig 5D and 5E).

The regulatory role of cGAS in FMDV replication was also investigated. The bone marrow-derived macrophages from cGAS^-/-^ mice were infected with FMDV. Similar to SVV and EV-A71 infection, IFN-β protein expression was significantly decreased in cGAS^-/-^ cells compared to that in WT cells during FMDV infection (Fig 5F), and higher titers of FMDV were detected in the cGAS^-/-^ cells compared to WT cells (Fig 5G).

The impact of the cGAS-STING signaling pathway on picornavirus replication was further assessed by overexpressing cGAS and/or STING. cGAS or STING can inhibit FMDV, SVV, and EV-A71 replication, and the co-expression of cGAS and STING played the most significant role in inhibiting viral replication (S5 Fig).

To further evaluate the impact of picornavirus-infection-induced mtDNA release on cGAS-mediated innate immune responses, the mtDNA-deficient PK-15 and HT-29 cells (referred to as ρ^0^) were generated by treatment of WT cells with a low-dose ethidium bromide (EB). The successful depletion of mtDNA from PK-15 and HT-29 cells was determined using qPCR (S6 Fig). The WT and ρ^0^ cells were infected with EV-A71, SVV, or FMDV, respectively, where the expression of IFN-β significantly decreased in the ρ^0^ cells compared to that in the WT cells (Fig 5H), suggesting that the mtDNA was critical for induction of IFN-β production during picornavirus infection. Furthermore, EV-A71, SVV, and FMDV yields were significantly higher in the ρ^0^ cells than in the WT cells (Fig 5I). Taken together, these results indicated that picornavirus infection triggered mtDNA release to activate cGAS-mediated antiviral response, and cGAS was critical for induction of IFN-β and ISGs expression during picornavirus infection.

### cGAS deficiency promoted EV-A71- and FMDV-induced mice death

**Fig 6:**
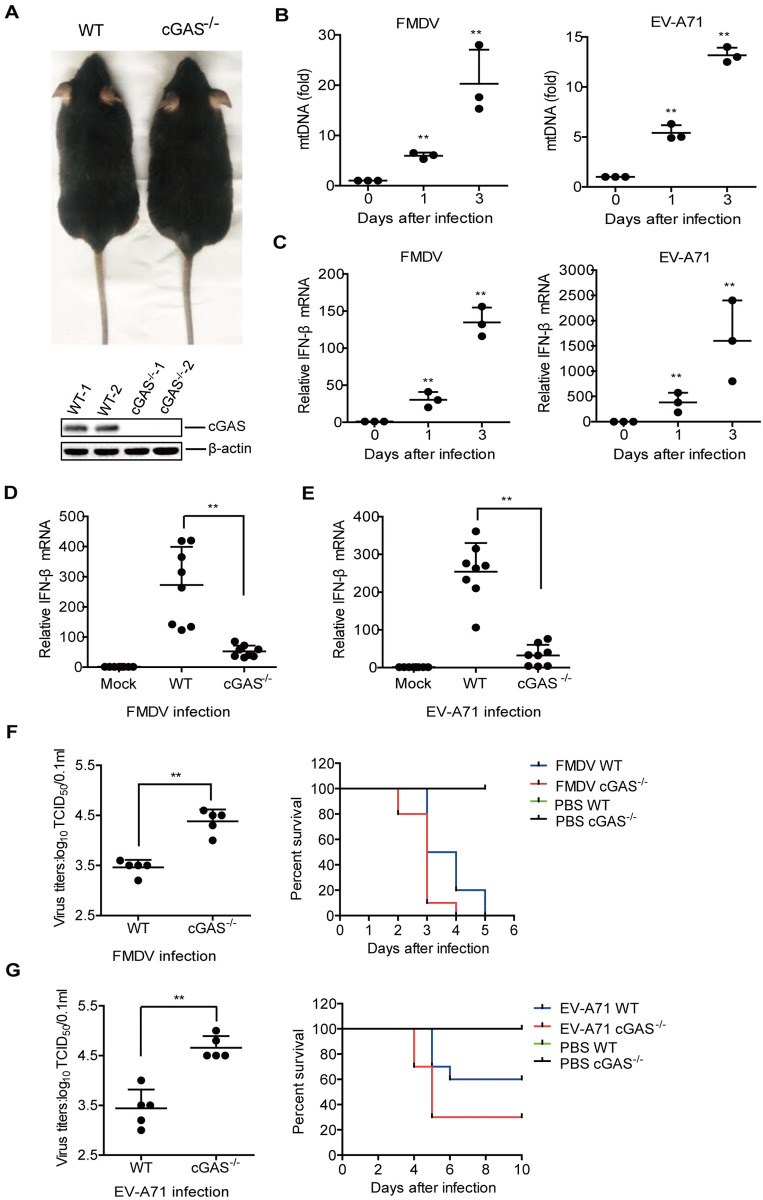
cGAS deficiency promoted EV-A71- and FMDV-induced mice death. (A) The 3-weeks-old WT and cGAS^-/-^ mice were showed, and the expression of cGAS in the WT and cGAS^-/-^ mice was detected by Western blotting. (B and C) The three-day-old WT mice were subcutaneously inoculated with FMDV (10^8^ TCID_50_) or EV-A71 (10^8^ TCID_50_) for 0, 1, or 3 d (n = 3/group), the mice carcasses without the head, tail, limbs, and viscera were collected for detection of mtDNA release (B) and IFN-β mRNA expression (C). (D and E) The three-day-old WT (n = 8) and cGAS^-/-^ (n = 8) mice were subcutaneously inoculated with FMDV (10^8^ TCID_50_) or EV-A71 (10^8^ TCID_50_). The IFN-β mRNA level in the carcasses without the head, tail, limbs, and viscera from FMDV-infected mice was measured and compared at 3 dpi by qPCR (D). The IFN-β mRNA level in the carcasses without the head, tail, limbs, and viscera from EV-A71-infected mice was measured and compared at 1 dpi by qPCR (E). FMDV (F) and EV-A71 (G) titers in the mice carcasses without the head, tail, limbs, and viscera were determined at 2 dpi by TCID_50_ assay. The mortality of WT (n = 10) and cGAS^-/-^ (n = 10) mice infected by FMDV (F) or EV-A71 (G) was determined, respectively. Error bars show standard deviation. **, *P*<0.01.

In the animal infection experiments, we determined that SVV did not induce mortality in mice. Thus, we investigated the function of cGAS in EV-A71 and FMDV infection in mice. The deficiency of cGAS protein expression in cGAS^-/-^ mice was confirmed by Western blotting (Fig 6A). In the WT mice infected by EV-A71 or FMDV, the mtDNA release was clearly observed in the mice carcasses without the head, tail, limbs, and viscera and increased as the infection progressed (Fig 6B). Similarly, IFN-β mRNA levels were significantly increased as the infection progressed (Fig 6C). The titers of EV-A71 and FMDV in the WT mice also gradually increased, confirming the correlation between mtDNA release and viral replication (S7 Fig).

Subsequently, the regulatory role of cGAS in EV-A71- or FMDV-induced innate immune responses was determined in mice. Our results indicated that the expression of IFN-β mRNA was significantly decreased in cGAS^-/-^ mice compared to that in WT mice during FMDV or EV-A71 infection (Fig 6D and 6E).

The titers of EV-A71 and FMDV were evaluated and compared in the WT and cGAS^-/-^ mice, suggesting that the viral titers were significantly enhanced in cGAS^-/-^ mice compared to that in WT mice during FMDV (Fig 6F, left) or EV-A71 (Fig 6G, left) infection. The impact of cGAS on EV-A71- and FMDV-induced mice mortality was evaluated as well. The WT mice infected with FMDV started to die at 3 dpi and all mice died by 5 dpi, while the cGAS^-/-^ mice infected by FMDV died more rapidly than FMDV-infected WT mice, which started to die at 2 dpi and all mice died by 4 dpi, indicating that cGAS deficiency accelerated the death of mice infected by FMDV (Fig 6F, right). EV-A71-infected WT mice survived 60%, while EV-A71-infected cGAS^-/-^ mice only survived 30% at 10 dpi, suggesting that cGAS deficiency resulted in higher mortality of the mice infected by EV-A71 (Fig 6G, right). Taken together, these results indicated that both FMDV and EV-A71 infection induced considerable mtDNA release in mice, and cGAS was essential for induction of type I IFN to suppress EV-A71 and FMDV infection *in vivo*.

### The impact of picornaviruses infection on cGAS and STING expression

**Fig 7:**
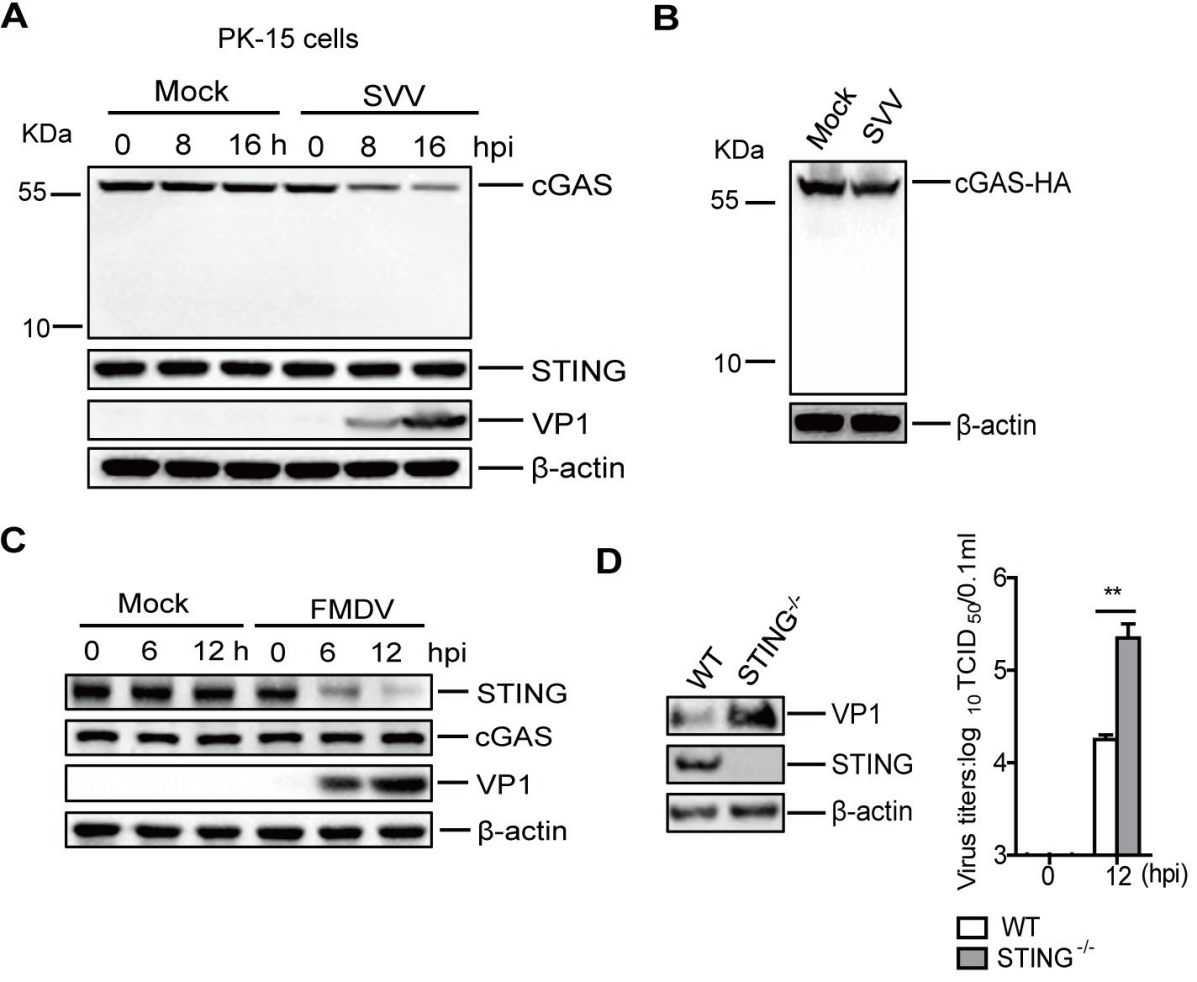
The impact of picornaviruses infection on cGAS and STING expression. (A) PK-15 cells were mock-infected or infected with SVV (MOI 1) for 0, 8, or 16 h. The expression of cGAS and STING proteins was detected by Western blotting. (B) HEK-293T cells were transfected with 1.5 μg of cGAS-HA expressing plasmids. At 24 hpt, the cells were mock-infected or infected with SVV (MOI 1) for 12 h. Expression of cGAS-HA protein was detected by Western blotting. (C) PK-15 cells were mock-infected or infected with FMDV (MOI 0.5) for 0, 6, or 12 h. Expression of cGAS and STING proteins was detected by Western blotting. (D) WT and STING^-/-^ PK-15 cells were infected with FMDV (MOI 0.5) for 12 h. The expression of FMDV VP1 protein was detected by Western blotting, and viral titers were determined by TCID_50_ assay. Error bars show standard deviation. **, *P*<0.01.

To further explore the potential role of cGAS in picornavirus infection, we investigated the state of cGAS and STING in picornavirus-infected cells. We found that EV-A71 infection did not affect the expression of cGAS and STING in both HeLa and HT-29 cells (S8 Fig). SVV infection induced a decrease of cGAS and did not affect the expression of STING (Fig 7A).

An anti-cGAS monoclonal antibody that detects the N terminal regions of cGAS was used in the experiments described above. To exclude the possibility that SVV infection cleaved cGAS at the C terminal region, HEK-293T cells were transfected with the C terminal HA-tagged cGAS expressing plasmids. At 24 h post-transfection (hpt), the cells were infected with SVV (MOI 1) for 12 h and then analyzed with an anti-HA antibody, and no cleaved bands were detected (Fig 7B).

The state of cGAS and STING in FMDV-infected cells was also investigated. FMDV infection induced the decrease of STING, and it did not affect the expression of cGAS in PK-15 cells (Fig 7C). The impact of STING on FMDV replication was then evaluated using STING^-/-^ cells, which showed that knockout of STING significantly enhanced FMDV replication (Fig 7D). Altogether, these results suggested that multiple antagonistic mechanisms can be used by picornaviruses to antagonize the antiviral effects of the cGAS-STING signaling pathway.

### SVV 2C protein was responsible for cGAS reduction

**Fig 8:**
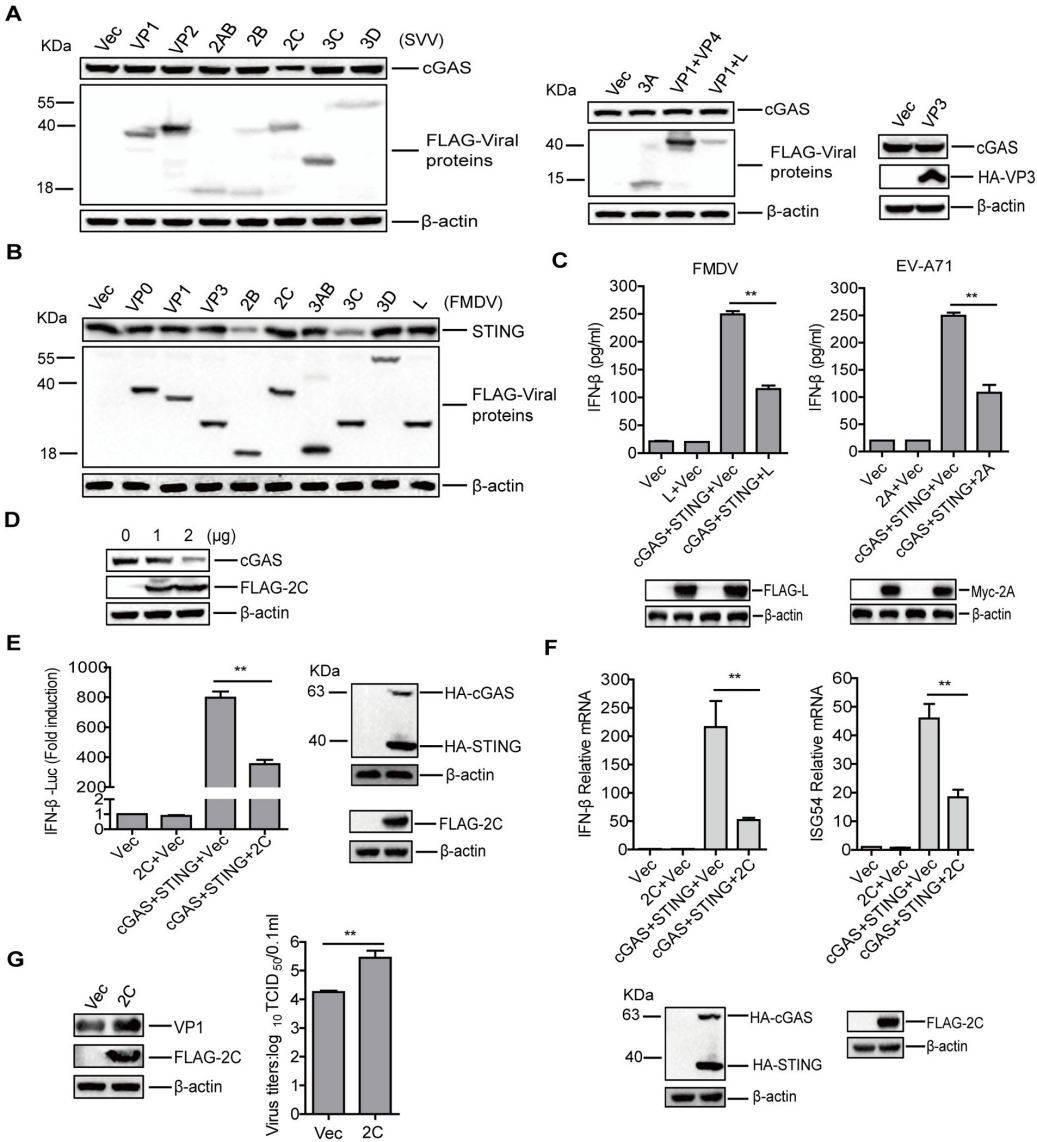
SVV 2C protein was responsible for cGAS reduction. PK-15 cells were transfected with 2 μg of plasmids expressing various SVV (A) and FMDV (B) proteins. The expression of endogenous cGAS or STING proteins was detected by Western blotting. (C) HEK-293T cells were transfected with 1 μg of empty vector or FLAG-L- and Myc-2A-expressing plasmids, and 1 μg of empty vector or HA-cGAS plus HA-STING expressing plasmids. At 24 hpt, the IFN-β protein expression level was determined by ELISA. (D) PK-15 cells were transfected with 0, 1, or 2 μg of plasmids expressing FLAG-2C protein. At 24 hpt, expression of endogenous cGAS protein was determined by Western blotting. (E and F) HEK-293T cells were transfected with 0.1 μg of empty vector or FLAG-2C-expressing plasmids, and 0.1 μg of empty vector or HA-cGAS plus HA-STING expressing plasmids, together with 0.1 μg of IFN-β-Luc, and 0.01 μg of pRL-TK plasmid. At 24 hpt, the promoter activation of IFN-β was determined by the dual-specific luciferase assay kit, and the expression of cGAS, STING, and 2C was confirmed by Western blotting (E). The mRNA expression levels of IFN-β and ISG54 were determined by qPCR assay (F).(G) PK-15 cells were transfected with 2 μg of empty vector or FLAG-2C-expressing plasmids. At 24 hpt, the cells were infected with SVV (MOI 1) for 12 h. Viral VP1 protein and titers were examined by Western blotting and TCID_50_ assay, respectively. Error bars show standard deviation. **, *P*<0.01.

The specific viral proteins that were responsible for inducing the reduction in cGAS or STING were further investigated by transfecting PK-15 cells with plasmids expressing different FLAG- or HA-tagged SVV or FMDV proteins. The expression of endogenous cGAS or STING protein was detected by Western blotting. Overexpression of SVV 2C protein significantly decreased the abundance of cGAS protein (Fig 8A), and overexpression of FMDV 2B or 3C^pro^ protein significantly decreased the abundance of STING protein (Fig 8B). FMDV L^pro^ and EV-A71 2A proteins can induce cleavage of eukaryotic translation initiation factor 4 G (eIF4G) [25,29,43]. Therefore, FMDV L^pro^ and EV-A71 2A proteins may antagonize cGAS-STING signaling pathway by blocking cap-dependent protein synthesis. Thus, the impact of FMDV L^pro^ and EV-A71 2A protein on the activation of cGAS-STING signaling pathway was detected. Overexpression of FMDV L^pro^ and EV-A71 2A significantly inhibited cGAS-STING-induced IFN-β protein secretion (Fig 8C). FMDV 2B and 3C^pro^ proteins antagonize host antiviral response that are triggered by several host-proteins [44]. Our results showed that FMDV 2B and 3C^pro^ proteins also inhibited cGAS-STING-induced IFN-β protein secretion (S9 Fig).

We further investigated the mechanism by which SVV 2C reduced cGAS protein expression. The 2C-induced reduction of endogenous cGAS was confirmed by performing a dose-dependent experiment (Fig 8D). The impact of SVV 2C on cGAS-mediated signal transduction and type I IFN production was also determined by carrying out luciferase reporter assay and qPCR assay. The results showed that cGAS-STING-induced IFN-β promoter activation (Fig 8E) and mRNA expression of IFN-β and ISG54 (Fig 8F) were inhibited by SVV 2C. To investigate a possible interaction between SVV 2C and cGAS, a coimmunoprecipitation experiment was performed. cGAS pulled down 2C in SVV-infected cells, and 2C reversely immunoprecipitated cGAS as well, suggesting that SVV 2C protein interacted with cGAS in the context of viral infection (S10 Fig).

SVV 2C performs antagonistic roles during SVV infection [45,46]. Here, we showed that SVV 2C induced a reduction of cGAS. However, the regulatory effect of 2C on SVV replication remains unknown. The replication of SVV in 2C overexpressing cells was then evaluated. As expected, overexpression of 2C significantly promoted SVV replication (Fig 8G). Taken together, these results indicated that overexpression of SVV 2C significantly decreased the expression of cGAS protein and enhanced SVV replication, and FMDV 2B, 3C^pro^, and L^pro^ proteins and EV-A71 2A protein inhibited the cGAS-STING signaling pathway.

### Identification of the pathways and functional sites in SVV 2C responsible for cGAS reduction

**Fig 9:**
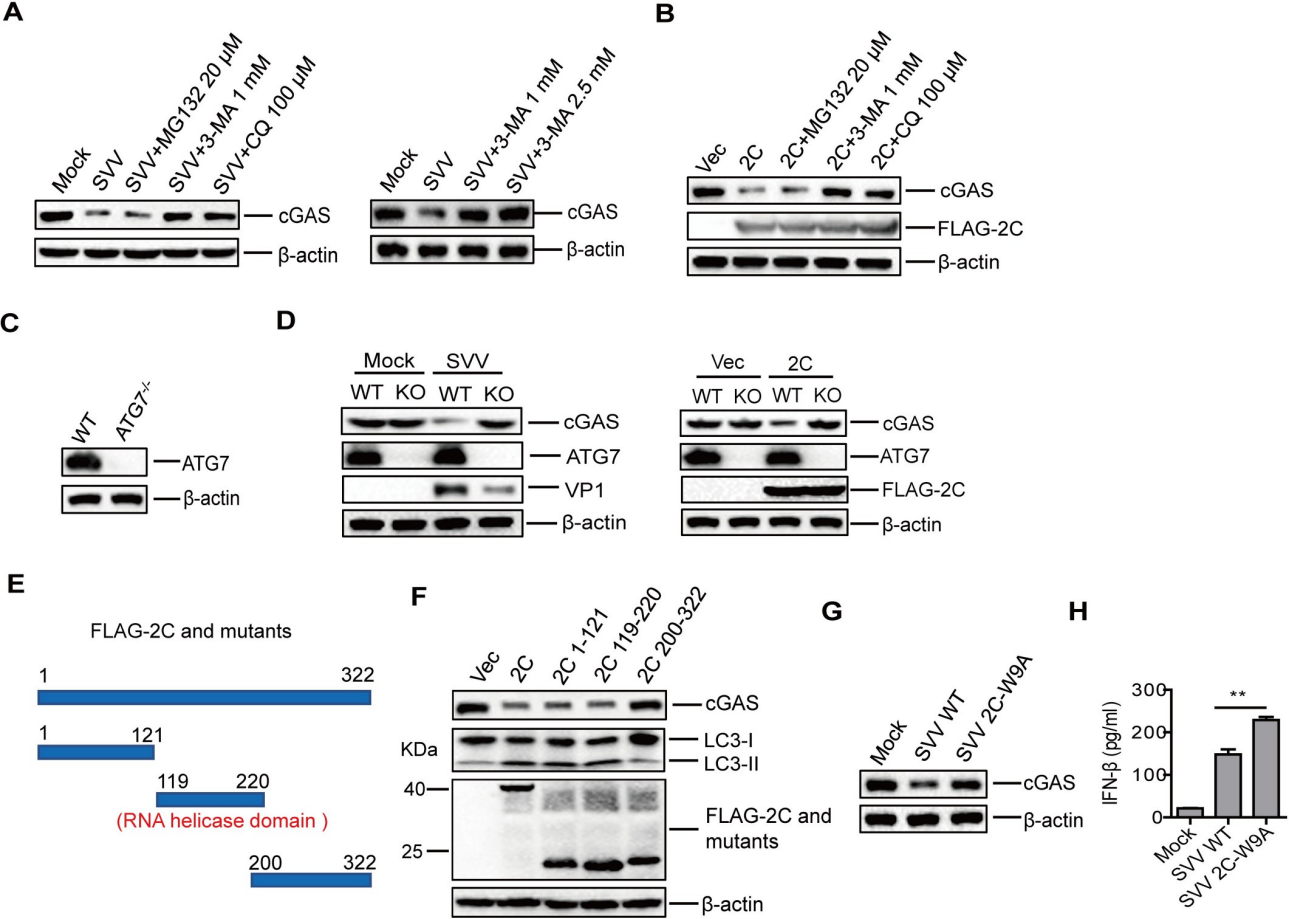
Identification of the pathways and 2C functional sites invovled in induction of cGAS reduction during SVV infection. (A) PK-15 cells were mock-infected or infected with SVV (MOI 1). At 1 hpi, the cells were maintained in the fresh medium in the presence or absence of MG132 (20 μM), 3-MA (1 or 2.5 mM), or CQ (100 μM). At 12 hpi, the expression of cGAS protein was determined by Western blotting. (B) PK-15 cells were transfected with 1.5 μg of FLAG vector or FLAG-2C-expressing plasmid. At 6 hpt, the cells were maintained in the fresh medium in the presence or absence of MG132 (20 μM), 3-MA (1 mM), or CQ (100 μM) for 18 h. Expression of cGAS protein was determined by Western blotting. (C) Expression of ATG7 protein in ATG7 WT and ATG7^-/-^ cells was detected by Western blotting. (D) ATG7 WT and ATG7^-/-^ cells were mock-infected or infected with SVV (MOI 1), or transfected with empty vector or FLAG-2C-expressing plasmids. At 12 hpi or 24 hpt, expression of cGAS, ATG7, and VP1 proteins was determined by Western blotting. (E) Schematic representation of a series of FLAG-tagged truncated 2C mutants. (F) PK-15 cells were transfected with empty vector, FLAG-2C- or the indicated FLAG-2C-mutant-expressing plasmids. At 24 hpt, expression of cGAS and LC3 proteins was determined by Western blotting. (G and H) PK-15 cells were mock-infected or infected with SVV WT (MOI 1) or SVV 2C-W9A (MOI 1) for 12 h. The expression of cGAS was detected by Western blotting (G). The IFN-β protein expression in the supernatant was determined by ELISA (H). Error bars show standard deviation. **, *P*<0.01.

Proteasomes and autophagy-lysosome pathways are two major intracellular protein degradation pathways in eukaryotic cells [47,48]. To evaluate whether these pathways were associated with SVV-induced reduction of cGAS, the autophagy inhibitors chloroquine diphosphate (CQ) and 3-MA and proteasome inhibitor MG132 were used to block these pathways during SVV infection, as described in our previous study [24]. PK-15 cells were mock-infected or infected with SVV and maintained in the presence or absence of the inhibitors. The expression of cGAS was detected by Western blotting. SVV-induced reduction of cGAS was inhibited by CQ and 3-MA, but not MG132 (Fig 9A). The impact of the inhibitors on 2C-induced reduction of cGAS was also evaluated. Treatment of 2C overexpressing cells with autophagy inhibitors (CQ or 3-MA) clearly reversed 2C-induced cGAS degradation, while incubation of the proteasome inhibitor did not affect the degradation of cGAS (Fig 9B). This indicated that both SVV- and 2C-induced reduction of cGAS were dependent on the autophagy pathway.

The autophagy-related protein autophagy-related 7 (ATG7) is associated with SVV-induced autophagy [49,50]. Therefore, we established the ATG7^-/-^ cells (Fig 9C) to block cellular autophagy for evaluating the impact of autophagy on the expression of cGAS. ATG7-WT and ATG7^-/-^ cells were mock-infected or infected with SVV (MOI 1) or transfected with an empty vector or the plasmids expressing 2C protein, and the collected cells were subjected to Western blotting analysis. Both SVV infection and overexpression of 2C induced the reduction of cGAS in the ATG7-WT cells, while it did not affect the expression of cGAS in the ATG7^-/-^ cells (Fig 9D). These results confirmed that ATG7 and the autophagy pathway were involved in SVV- and 2C-induced cGAS reduction.

To further confirm the functional domains of 2C that were essential for induction of cGAS reduction, NCBI functional analysis tools (https://www.ncbi.nlm.nih.gov/Structure/cdd/wrpsb.cgi) were used to predict whether 2C includes any functional domains. The results showed that 2C region encompassing amino acids 119–220 included a putative RNA helicase domain. Therefore, three SVV 2C truncation mutants based on the RNA helicase domain were constructed (Fig 9E). FLAG-2C or the truncated FLAG-2C mutants expressing plasmids were transfected into PK-15 cells. The abundance of cGAS was then detected by Western blotting. The 1–121 and 119–220 amino acid regions of 2C contributed to the reduction of cGAS, while the 200–322 region had no effect on cGAS expression (Fig 9F). These results indicate that both the N terminal region and the RNA helicase domains of 2C were responsible for the 2C-induced reduction of cGAS protein. In addition, we found that both the N terminal region and RNA helicase domain of 2C induced autophagy (Fig 9F), consistent with the regions in 2C responsible for cGAS reduction.

Subsequently, a series of truncated or site-mutation mutants constructs of SVV 2C were generated and the functional sites in SVV 2C responsible for cGAS reduction were further identified. The results showed that the 9th and 153rd aa sites in 2C were critical for inhibitory function against cGAS expression (S11 Fig). To further confirm the role of these sites to decrease cGAS expression, three recombinant SVVs were tried to be rescued by introducing single-site mutation W9A or L153A, or double-site mutation of W9A-L153A into 2C, as described previously [51]. The recombinant wildtype SVV was used as the parental virus (SVV WT). The W9A mutant SVV was successfully rescued (named SVV 2C-W9A). However, the L153A and W9A-L153A mutant viruses could not be rescued, which suggested that the 153rd site of 2C was essential for SVV replication. The expression of cGAS and IFN-β protein in SVV WT- and SVV 2C-W9A-infected cells was evaluated and compared, which showed that the expression of cGAS (Fig 9G) and IFN-β (Fig 9H) was significantly enhanced in SVV 2C-W9A-infected cells compared to that in SVV WT infected cells, confirming the inhibitory effect of the 9th site in 2C on cGAS expression. Taken together, these results indicated that SVV 2C protein decreased the abundance of cGAS via the autophagy pathway, and W9 and L153 amino acid sites of 2C were responsible for cGAS reduction.

### EV-A71, CA16, and EMCV 2C protein antagonized cGAS-STING pathway by impairing the interaction between STING and TBK1

**Fig 10:**
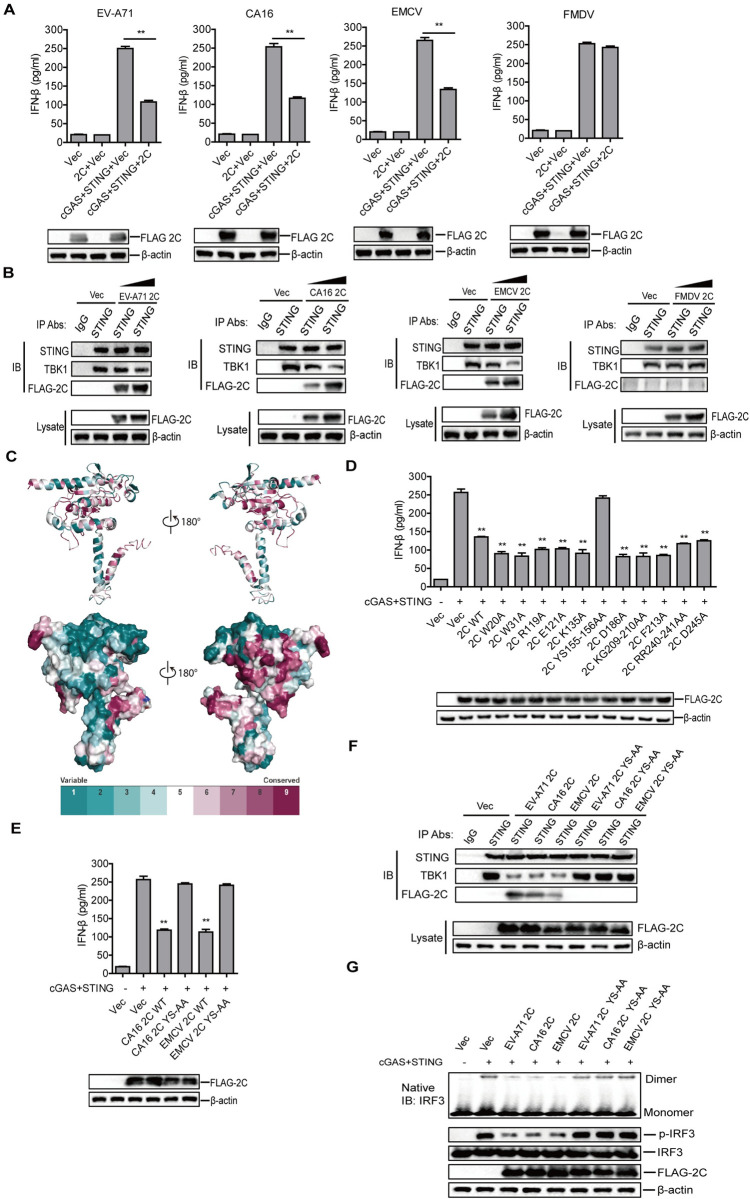
EV-A71, CA16, and EMCV 2C proteins antagonized cGAS-STING signaling pathway activation by impairing the interaction between STING and TBK1. (A) HEK-293T cells were transfected with 1 μg of empty vector or the indicated FLAG-2C-expressing plasmids, along with 1 μg of empty vector or HA-cGAS plus HA-STING expressing plasmids. At 24 hpt, the IFN-β protein amount in the supernatant was determined by ELISA, and the expression of 2C was confirmed by Western blotting. (B) STING-HEK-293T cells were transfected with increasing amount (0, 3, or 6 μg) of the indicated FLAG-2C-expressing plasmids. At 24 hpt, cells were transfected with poly(dA:dT) (2 μg/ml) for 12 h. The cells lysates were immunoprecipitated with anti-STING antibody. The antibody-antigen complexes were detected using anti-STING, TBK1, and FLAG antibodies, respectively. (C) Schematic representation of the structure and conserved functional sites in EV-A71 2C protein. The redder the color was, the more conservative the sites were. (D, E) HEK-293T cells were transfected with 1 μg of empty vector or EV-A71 (D), CA16 (E), and EMCV (E) FLAG-2C- or FLAG-2C mutants-expressing plasmids, along with 1 μg of empty vector or HA-cGAS plus HA-STING expressing plasmids. At 24 hpt, the IFN-β protein amount in the supernatant was determined by ELISA, and the expression of 2C was confirmed by Western blotting. (F) STING-HEK-293T cells were transfected with 6 μg of empty vector, FLAG-2C- or FLAG-2C mutants-expressing plasmids. At 24 hpt, cells were transfected with poly(dA:dT) (2 μg/ml) for 12 h. The cells lysates were immunoprecipitated with anti-STING antibody. The antibody-antigen complexes were detected using anti-STING, TBK1, and FLAG antibodies, respectively. (G) HEK-293T cells were transfected with 1 μg of empty vector, FLAG-2C- or FLAG-2C mutants-expressing plasmids, and 1 μg of HA-cGAS plus HA-STING expressing plasmids. At 24 hpt, expression of IRF3, p-IRF3, and FLAG-2C protein was determined by Western blotting. The IRF3 dimerization was detected using native PAGE. Error bars show standard deviation. **, *P*<0.01.

Although EV-A71 infection did not affect the expression of cGAS and STING, we also detected the impact of EV-A71 2C protein on the activation of cGAS-STING signaling pathway. EV-A71 2C significantly inhibited cGAS-STING-induced IFN-β production in HEK-293T cells (Fig 10A). The impact of 2C protein of other picornaviruses on the activation of cGAS-STING pathway was investigated subsequently. CA16 and EMCV 2C, but not FMDV 2C antagonized cGAS-STING-induced IFN-β production (Fig 10A). The interaction between STING and TBK1 is essential for type I IFN production. To explore by which picornavirus 2C inhibited activation of cGAS-STING pathway, we detected the interaction between STING and TBK1. STING-HEK-293T cells were transfected with poly(dA:dT) along with empty vector or FLAG-2C-expressing plasmid. At 24 hpt, the cells lysates were immunoprecipitated with anti-STING antibody and analyzed by Western blotting. STING pulled down TBK1, and EV-A71, CA16, and EMCV 2C, but not FMDV 2C, interacted with STING and inhibited the interaction between STING and TBK1 in a dose-dependent manner (Fig 10B), suggesting that the interaction between 2C and STING reduced the interaction between STING and TBK1. These results indicated that EV-A71, CA16, and EMCV 2C were responsible for inhibition of the cGAS-STING signaling pathway activation by impairing the interaction between STING and TBK1.

To identify the functional sites of 2C that were essential for inhibition of the cGAS-STING signaling pathway, the iterative threading assembly refinement (I-TASSER) server [52] and consurf [53] analysis tools were used to predict structure and conserved functional sites of 2C, respectively. The structure of EV-A71, CA16, and EMCV 2C are highly similar. Therefore, only the structure diagram of EV-A71 2C protein is presented in Fig 10C, and the conserved functional sites W20, W31, R119, E121, K135, YS155-156, D186, KG209-210, F213, RR240-241, and D245 were selected for further study. A series of EV-A71 2C site mutants were constructed, and the impact of EV-A71 2C mutants on activation of the cGAS-STING signaling pathway was evaluated and compared. EV-A71 2C YS155-156 amino acids (named EV-A71 2C YS-AA) mutant did not affected cGAS-STING-induced IFN-β production, while 2C WT and other 2C mutants significantly inhibited the activation of the cGAS-STING signaling pathway (Fig 10D).

The amino acid sequences of 2C proteins of EV-A71, EMCV, CA16, and FMDV were compared and analyzed. Similar to EV-A71 2C, EMCV and CA16 2C also contain YS sites, while FMDV 2C does not have this conserved YS sites (S12 Fig). This explained why FMDV 2C failed to inhibit cGAS-STING-induced IFN-β production. We next constructed EMCV and CA16 2C YS-AA mutants, and evaluated the impact of these mutants on activation of cGAS-STING pathway. EMCV and CA16 2C WT considerably inhibited cGAS-STING-induced IFN-β production, while 2C YS-AA mutants abrogated activation of the cGAS-STING signaling pathway (Fig 10E).

The effect of 2C YS-AA mutants on the interaction between STING and TBK1 was further evaluated. EV-A71, CA16, and EMCV 2C interacted with STING and inhibited the interaction between STING and TBK1, while EV-A71, CA16, and EMCV 2C YS-AA mutants did not interact with STING and abrogated the reduction of interaction between STING and TBK1 (Fig 10F), suggesting that 2C YS was the critical sites responsible for 2C-STING interaction, and the binding of 2C to STING directly blocked the interaction between STING and TBK1.

TBK1 mediates phosphorylation and dimerization of IRF3, resulting in the activation of IRF3 and the induction of type I IFNs [54]. Therefore, we further investigated the impact of 2C and 2C YS-AA mutants on IRF3 phosphorylation and dimerization. The results showed that EV-A71, EMCV, and CA16 2C also inhibited cGAS-STING induced IRF3 phosphorylation and dimerization, while 2C YS-AA mutants did not affect it (Fig 10G). Taken together, these results indicated that EV-A71, EMCV, and CA16 2C impaired the interaction between STING and TBK1, resulting in the inhibition of IRF3 phosphorylation and dimerization, and the YS sites in 2C were responsible for this inhibitory effect.

## Discussion

It is generally accepted that cGAS senses viral and intracellular DNA to activate STING-TBK1-IRF3 to induce IFNs production. In recent years, increasing evidence has suggested that RNA viruses, such as DENV, ZIKV, EMCV, and IAV, can activate the cGAS-STING signaling pathway during viral infection [15–17,55]. Here, we investigated for the first time the functions of cGAS-STING signaling pathway during picornavirus infection and provided evidence that picornavirus infection activates cGAS-mediated signal transduction by mtDNA. IFA showed that EV-A71 infection induced high amounts of mtDNA release, while SVV and FMDV infection induced lower levels of mtDNA release, differences which may be due to the cells or time points of infection.

Mitochondria are a widely studied organelle since they are major producer of intracellular ROS, which has been linked to the cause of aging and other diseases [56]. In virus infection, the virus-mitochondria interaction plays important roles in viral pathogenic mechanisms [57–59]. Our results showed that EV-A71 infection induced mitochondrial ΔΨm reduction in HeLa cells, which is in accordance with reports of mitochondrial damage in RD cells caused by EV-A71 infection [60]. mPTP is a protein complex that is proposed to span the inner and outer mitochondrial membranes to facilitate the loss of the inner membrane potential, swelling, and eventual rupture of the organelle [61]. There are only a few reports in the literature that describe the impact of viral infection on mPTP opening. For instance, hepatitis E virus (HEV) infection induces mPTP opening and mitochondrial dysfunction to damage hepatocytes [62]; hepatitis B virus (HBV) infection induces apoptosis through modulation of mPTP opening [63]. Here, we found for the first time that EV-A71, SVV and FMDV infection regulated mitochondrial ΔΨm, ROS, Ca^2+^, and mPTP opening to damage mitochondria, resulting in the release of mtDNA to regulate innate immune responses.

The mitochondrial outer membrane can be permeabilized by Bax and Bak, resulting in the release of mtDNA to activate innate immune signaling pathways [64]. Many viruses trigger mtDNA through different mechanisms. Severe fever with thrombocytopenia syndrome virus (SFTSV) induces Bak/Bax-dependent mtDNA release [13]. Dengue virus (DENV) infection induces the activation of cGAS-mediated signaling pathway by triggering the release of mtDNA, but the mechanisms involved remain unknown [14]. Furthermore, influenza virus M2 and EMCV 2B proteins trigger the release of mtDNA into the cytosol in a MAVS-dependent manner [16]. We determined that picornavirus 2B protein promotes mtDNA release through a VDAC1-dependent manner during picornavirus infection. VDAC1 is a newly discovered outer membrane channel involved in mtDNA release [41]. In the present study, SVV infection induced VDAC1- and Bak/Bax-dependent mtDNA release, EV-A71 and FMDV infection induced VDAC1-dependent but Bak/Bax-independent mtDNA release, revealing multiple mtDNA release mechanisms arising from the infection of different picornaviruses. In addition, the cytosolic mtDNA induced by SVV, EV-A71, or FMDV infection can bind to cGAS, resulting in the activation of the cGAS-STING signaling pathway and type I IFN expression. The *in vitro* and *in vivo* experiments firmly confirmed that the activated cGAS inhibited SVV, EV-A71, and FMDV replication by regulation of type I IFN production. These results revealed a common mechanism by which picornavirus infection triggers the cGAS-STING signaling pathway activation, and it is different from the mechanisms utilized by DENV, SFTSV, or influenza A virus [14,16]. EV-A71 infection induced the loss-of cap-dependent protein synthesis and antagonized host innate immune responses through multiple pathways [65]. Therefore, EV-A71 infection does not induce IFN-β mRNA in RD and Hela cells, but it promotes IFN-β mRNA in the HT-29 cells [66]. Previous studies have shown that EV-A71 can induce high levels of IFN-β mRNA [58,59], but our data showed that EV-A71 infection induced low levels of IFN-β mRNA, which may be due to different calculation methods or different MOI of EV-A71.

The impact of the cGAS-STING signaling pathway on viral replication has been described for several RNA viruses. In some, STING is not essential for induction of IFN expression, but essential for inhibiting RNA virus replication through restriction of viral protein synthesis [67,68]. Studies have indicated that cGAS or STING inhibits DENV, ZIKV, Japanese encephalitis virus, West Nile virus, and influenza A virus replication [69,70]. In the present study, we determined the antiviral role of cGAS and STING against SVV, EV-A71, and FMDV replication. These results indicated that the cGAS-STING signaling pathway plays important antiviral roles against picornavirus infection.

The expression of cGAS protein directly affects cGAS-mediated pathway signal transduction [71,72]. To date, the regulation of cGAS protein expression through various mechanisms has been reported for different viruses. For example, DENV directly cleaves cGAS through the protease complex NS2B3, ZIKV induces the cleavage of cGAS through stabilization of caspase-1 by viral NS1 protein [14,15], and vaccinia virus deregulates mTOR, resulting in enhanced degradation of cGAS [73]. In addition, cellular apoptotic caspases induce the cleavage of cGAS [74], and host TRIM14 protein contributes to cGAS degradation [75]. Here, we determined that SVV infection induced the reduction of cGAS protein, while FMDV and EV-A71 did not affect the expression of cGAS, which suggest that the reduction of cGAS induced by SVV is specific. In addition, we found that FMDV infection induced the reduction of STING protein; EV-A71 infection did not affect the expression of cGAS and STING, but blocked the recruitment of TBK1 to STING, revealing different antagonistic mechanisms used by different picornaviruses to impair cGAS- and STING-mediated antiviral functions.

SVV 2C is an approximately 40-kDa protein, which is involved in cell apoptosis and inhibition of innate immune responses [46,76]. Our results demonstrated that SVV 2C protein induces the degradation of cGAS in an autophagy pathway-dependent manner, which is similar with the strategy used by Chikungunya virus (CHIKV) for degradation of cGAS [55]. Autophagy is always associated with the host’s innate immune response [77]. Our results revealed that the activation of autophagy plays an important regulatory role in SVV-mediated innate immune responses. For the first time, we showed that the 9th and 153rd sites in the N terminal region and the deductive RNA helicase domain of 2C were responsible for cGAS reduction, highlighting the importance of these sites.

Our results also indicated that overexpression of 2C promoted SVV replication, and multiple functions of 2C might have contributed to the enhanced viral replication. For example, SVV 2C-induced intracellular apoptosis and reduction of RIG-I may be important for SVV replication [46,76]. Here, the 2C-induced reduction of cGAS is determined to be another critical factor that promotes SVV replication in the infected cells.

2C is a multifunctional and highly conserved nonstructural protein that plays a complex, yet poorly understood role in the natural history of picornaviruses. EV-A71 2C structure consists of three subdomains: an ATPase domain, a zinc finger, and a long protruding C-terminal α helix [78]. The 116–329 aa in 2C exhibits potent ATPase activity, and any substitution of K135A, R240, or R241 can abort ATPase activity [78]. Any mutation of the zinc coordinators C270A, C281A, or C286A can result in insoluble 2C [78]. EV-A71 2C is involved in host innate immune responses by regulating IKKα, IKKβ, or p65 protein [27,28]. CA16 2C regulates autophagy to enhance viral replication [79]. EMCV 2C protein antagonizes IFN-β signaling pathway through its interaction with MDA5 [80]. Here, we determined that EV-A71, CA16, and EMCV 2C antagonize cGAS-STING signaling pathway through its interaction with STING. The detailed analysis showed that the K135A and R240-R241 mutants lacking ATPase activity still play an important role in inhibition of cGAS-STING signaling pathway activation, suggesting that the ATPase activity of 2C was not essential for inhibition of cGAS-STING-induced IFN-β production. Furthermore, we showed that the YS sites in 2C were responsible for suppression of cGAS-STING signaling pathway activation, highlighting the importance of these sites and being used as a potential therapeutic target for combating picornaviral diseases.

**Fig 11:**
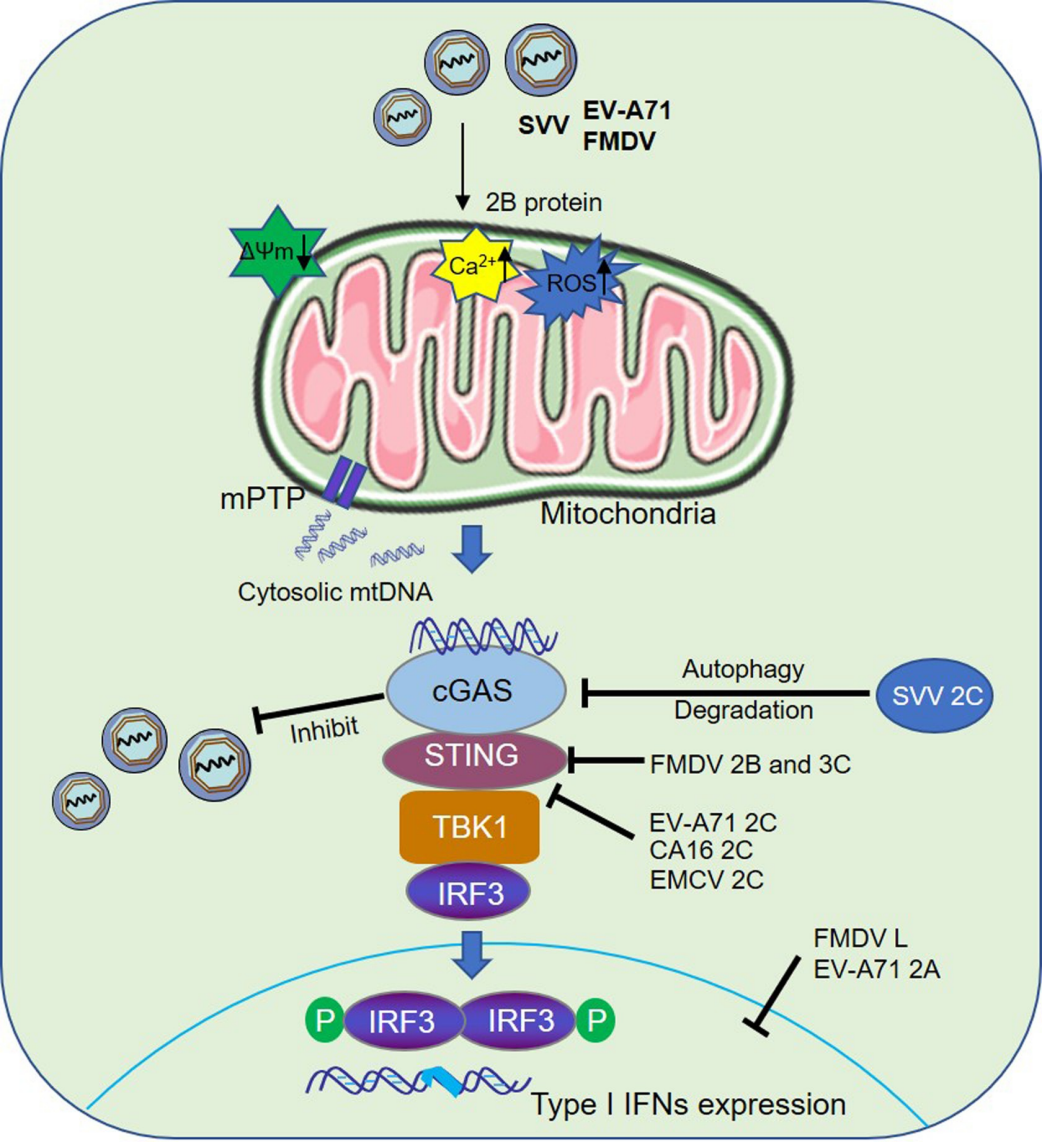
Schematic representation of the model of cGAS-mediated antiviral function in EV-A71, SVV, and FMDV replication. In this model, cytosolic mtDNA released after EV-A71, SVV, and FMDV infection binds to cGAS to activate cGAS-mediated signal transduction, resulting in the antiviral responses. Virus immune evasion strategies include SVV 2C induced the reduction of cGAS by activation of autophagy and FMDV 2B and 3C^pro^ proteins which inhibit STING expression to block the antiviral response. Furthermore, EV-A71, CA16, and EMCV 2C can antagonize activation of the cGAS-STING signaling pathway by impairing the interaction of STING with TBK1, while FMDV L^pro^ and EV-A71 2A proteins inhibit cGAS-STING-induced IFN-β protein expression.

In conclusion, our results showed that picornavirus infection leads to activation of the cGAS-mediated signaling pathway by triggering the release of mtDNA via mPTP, and that the activated cGAS plays an important antiviral role by regulating type I IFN expression. For the first time, we also describe novel antagonistic mechanisms by which SVV 2C degrades the expression of cGAS protein to inhibit cGAS-induced antiviral effect, and by which EV-A71, CA16, and EMCV 2C impair the interaction between STING and TBK1 to block the activation of the cGAS-STING signaling pathway. In addition, FMDV L^pro^ and EV-A71 2A proteins inhibited cGAS-STING-induced IFN-β protein expression (Fig 11).

## Materials and methods

### Ethics statement

All animals were handled strictly with good animal practice according to the Animal Ethics Procedures and Guidelines of the People’s Republic of China, and the study was approved by the Animal Ethics Committee of Lanzhou Veterinary Research Institute of the Chinese Academy of Agricultural Sciences (Licence no. SYXK (GAN) 2010–003).

### Cells

HEK-293T cells, STING-HEK-293T cells, PK-15 cells, HeLa cells, RD cells, and HT-29 cells were cultured in Dulbecco’s modified Eagle medium (Gibco) supplemented with 10% heat-inactivated fetal bovine serum (Gibco) and maintained at 37°C (5% CO_2_). STING knockout PK-15 cells were provided by X.D. Qin (Lanzhou Veterinary Research Institute). The stably expressing STING HEK-293T cell lines (STING-HEK-293T) were stored in our laboratory. Mouse bone marrow-derived macrophage (BMDM) from WT and cGAS^-/-^ mice were isolated and cultured as described previously [81].

### Mice

cGAS^-/-^ mice (C57BL/6) were purchased from Cyagen Biosciences, China and maintained in the specific pathogen-free (SPF) animal facility of Lanzhou Veterinary Research Institute with free access to food and water. Mouse experimental work was performed using 3-days-old suckling mice, and was age- and sex-matched in each experiment. The mice carcasses without the head, tail, limbs, and viscera were collected for detection of mtDNA and IFN-β expression.

### Viruses and infection

The SVV strain was isolated from cases of SVV in Guangdong Province, China and stored in our laboratory [82], and virus propagation and titration were performed by using IBRS-2 cells. FMDV type O strain O/BY/CHA/2010 was used for the viral challenge [83] which was propagated and titrated using BHK-21 cells. The EV-A71 strain H (VR-1432) stored in our laboratory was used for the viral challenge [83,84], and virus propagation and titration were performed by using RD cells. The 50% tissue culture infectious dose (TCID_50_) was calculated by Reed and Muench method. All virus stocks were stored at −80°C until use. Viral infection experiments were carried out as described previously [85].

### Titration of FMDV and EV-A71 from mice

FMDV and EV-A71 in the mice carcasses without the head, tail, limbs, and viscera were isolated as described previously [86]. Briefly, to quantify the number of the virus particles, the mice carcasses were weighed and homogenized using disposable tissue grinders (VWR, Radnor, PA) in Dulbecco’s modified Eagle medium (DMEM, Gibco) supplemented with 1% penicillin-streptomycin-neomycin antibiotic mixture, 2.5 μg/mL fungizone, and 1% L-Glutamax (ThermoFisher, Waltham, MA). Titration of FMDV and EV-A71 was performed using DMEM media supplemented with 1% penicillin-streptomycin-neomycin antibiotic mixture, 2.5 μg/mL fungizone, and 1% L-Glutamax, as described previously [86,87].

### Plasmids and antibodies

IFN-β promoter luciferase reporter plasmids and the pRL-TK *Renilla* luciferase reporter plasmid were prepared in our laboratory as described previously [45]. The SVV, FMDV, and EV-A71 full-length viral cDNAs were inserted into the p3xFLAG-CMV-7.1 vector (Sigma-Aldrich, St. Louis, MO, USA) to construct plasmids expressing FLAG-tagged viral proteins. SVV VP3 cDNA was inserted into PcDNA3.1-HA vector (Sigma-Aldrich) to construct plasmids expressing HA-tagged VP3 protein. EV-A71 2A cDNA was inserted into PcDNA3.1-Myc vector (Sigma-Aldrich) to construct plasmids expressing Myc-tagged 2A protein. A series of FLAG-tagged truncated 2C constructs were prepared in our laboratory through site-directed mutagenesis PCR. HA-cGAS- and HA-STING-expressing plasmids were constructed by inserting the CDS of cGAS or STING into PcDNA3.1-HA vector plasmid. All the constructed plasmids were analyzed and verified by DNA sequencing. The plasmids were transfected into the cells using Lipofectamine 3000 reagent (Invitrogen) according to the manufacturer’s protocol.

The commercial antibodies used in this study included an anti-FLAG monoclonal antibody (Santa Cruz Biotechnology, Dallas, TX, USA), anti-FLAG polyclonal antibody (Sigma-Aldrich), anti-HA monoclonal antibody (Thermo Scientific, Waltham, MA, USA), anti-cGAS monoclonal antibody (Santa Cruz Biotechnology), anti-STING monoclonal antibody (Cell Signaling Technology, Beverly, MA, USA), anti-p-STING (S366) monoclonal antibody (Cell Signaling Technology), anti-IFI16 monoclonal antibody (Cell Signaling Technology), anti-TBK1 monoclonal antibody (Cell Signaling Technology), anti-IRF3 monoclonal antibody (Cell Signaling Technology), anti-p-IRF3 monoclonal antibody (Cell Signaling Technology), anti-dsDNA monoclonal antibody (Abcam, Cambridge, MA, USA), anti-ATG7 polyclonal antibody (ABclonal Technology Co., Ltd, Wuhan, China), anti-Tom20 monoclonal antibody (ABclonal), and anti-β-actin monoclonal antibody (Thermo Scientific), anti-Bax monoclonal antibody (ABclonal), anti-PPID polyclonal antibody (ABclonal), anti-EV-A71 3C polyclonal antibody (ABclonal), anti-SVV VP1 polyclonal antibody was prepared in our laboratory [45], anti-FMDV VP1 polyclonal antibody was prepared in our laboratory [24], and anti-2C polyclonal antibody was produced in rabbits by immunization with SVV 2C protein.

### Mitochondrial membrane potential

Mitochondrial membrane potential was detected using mitochondrial membrane potential assay kit (Beyotime, Shanghai, China), according to the manufacturer’s instructions. Briefly, the cells were mock-infected or infected with picornavirus and collected and stained with JC-1 (1:200) at 37°C for 30 min. JC-1 fluorescence was tested at 514–529 nm (green fluorescence, JC-1 monomers) and 585–590 nm (red fluorescence, JC-1 aggregates) using a spectrofluorometer (Thermo Scientific). JC-1 fluorescence images were visualized using a Nikon Eclipse 80i fluorescence microscope. The images were captured using NIS Elements F 2.30 software. The occurrence of JC-1 green fluorescence indicates mitochondrial depolarization.

### Detection of mPTP opening

mPTP opening was detected using an mPTP assay kit (Beyotime, Shanghai, China), according to the manufacturer’s instructions. Briefly, the mock-infected or infected cells were collected and stained with calcein AM staining solution with CoCl_2_ or calcein AM staining solution with CoCl_2_ and lonomycin at 37°C for 45 min. Lonomycin is a calcium ionophore which is used as a positive control to open mPTP. The fluorescence was tested at 494–517 nm using a spectrofluorometer (Thermo Scientific).

### Detection of mitochondrial reactive oxygen species (ROS)

Mitochondrial ROS were detected using the mitoSOX red mitochondrial superoxide indicator (Yeasen Biotech, Shanghai, China). This fluorogenic dye can enter the mitochondria of living cells and is oxidized by superoxide anions. Cells mock-infected and infected viruses were collected and stained with mitoSOX (2 μM) at 37°C for 10 min, followed by a wash with PBS. The fluorescence was tested at 510–580 nm using a spectrofluorometer (Thermo Scientific).

### Detection of mitochondrial Ca^2+^ concentration

Mitochondrial Ca^2+^ was detected using indicator Rhod-2 AM (Abcam, Cambridge, MA, USA), as described previously [88]. Mock-infected and virus infected cells were collected and stained with Rhod-2 AM (1 μM) at 37°C for 60 min. The fluorescence was tested at 552–581 nm using a spectrofluorometer (Thermo Scientific).

### Coimmunoprecipitation and Western blotting analysis

PK-15 cells cultured in 10-cm dishes were transfected with the indicated plasmids, and mock-infected or infected with viruses for the indicated time. The collected cells were lysed and immunoprecipitated as described previously [89].

The target proteins were analyzed by 10% sodium dodecyl sulfate-polyacrylamide gel electrophoresis (SDS-PAGE) and transferred to an Immobilon-P membrane (Millipore, Bedford, MA, USA), which was blocked with 5% skim milk in TBST and treated with appropriate primary (1:1000) and secondary antibodies (1:5000). The antibody-antigen complexes were visualized using enhanced chemiluminescence detection reagents (Share-bio Biotechnology, Shanghai, China).

### Native PAGE

The dimerization of IRF3 was analyzed by native PAGE, as described previously [90]. Briefly, cells were collected and lysed using RIPA lysis buffer (Solarbio, Beijing, China) for 30 min and 16 μg of cell lysates were used for native PAGE. 7.5% native-PAGE gels were made without SDS. The gels were pre-run with 25 mM Tris and 192 mM glycine, pH 8.4, and 1% deoxycholate (DOC) for 30 min at 40 mA. After that, samples treated with native sample buffer were size-fractionated by electrophoresis for 60 min at 25 mA and then ransferred to PVDF membranes for immunoblot analysis.

### IFN-β reporter assay

HEK-293T cells cultured in 24-well plates were co-transfected with 0.1 μg/well of IFN-β-Luc, 0.01 μg/well of pRL-TK Renilla luciferase reporter plasmid, and 0.1 μg/well of FLAG-2C-, HA-cGAS-, or HA-STING-expressing plasmids. Lipofectamine 3000 (Invitrogen) was used as the transfection reagent. At 24 hpt, the cells were lysed and analyzed by the dual-specific luciferase assay kit (Promega) according to the manufacturer’s instructions.

### Establishment of knockout cell lines using CRISPR/Cas9 system

The cGAS, PPID, Bax, and ATG7 knockout cell line was established as described previously [91]. The small guide RNAs (sgRNA) target human cGAS, PPID, Bax and porcine PPID, Bax, ATG7, were designed using the online CRISPR design tool (http://crispr.mit.edu/). The sgRNA was inserted into the pLentiCRISPR plasmid with the puromycin selection gene. The constructs were transfected to cells using Lipofectamine 3000. Cells were selected by puromycin (2 μg/mL) to obtain stable knockout cells. After confirmation of the activity of the designed sgRNA using the T7 Endonuclease I (NEB), the KO cell lines were confirmed by Western blotting. Control cells were transfected with the empty vector. The sgRNA sequences are as follows. Human cGAS: GGCCGCCCGTCCGCGCAACT(GGG) [92], human PPID: GGCGACTTCACCAACCACAA [93], human Bax: TCGGAAAAAGACCTCTCGGG, porcine ATG7: CAACTTGAGATTGAGGTCCACGG, porcine PPID: CCGCTTGTGTACCTGGACGT, porcine Bax: GCTGACGGCAACTTCAACTG.

### RNA extraction and quantitative PCR (qPCR)

Total RNAs from the samples were extracted using TRIzol reagent (Invitrogen). cDNAs were synthesized using the Moloney murine leukemia virus reverse transcriptase (Promega, Madison, WI, USA) and random hexamer primers (TaKaRa, Shiga, Japan). The expression of viral and host mRNA was detected using the cDNA, Mx3005P qPCR System (Agilent Technologies, Palo Alto, CA, USA), and SYBR Premix Ex Taq reagents (TaKaRa, Dalian, China). The *GAPDH* gene was used as an internal control. The relative expression of mRNA was calculated based on the comparative cycle threshold (CT) (2^−ΔΔCT^) method [94]. The primers targeting mouse genes were as described previously [16]. The qPCR primers sequences are as follows:

human IFN-β-F: GACATCCCTGAGGAGATTAAG, R: ATGTTCTGGAGCATCTCATAG;
human ISG54-F: ACGGTATGCTTGGAACGATTG, R: AACCCAGAGTGTGGCTGATG;
human GAPDH-F: CGGGAAGCTTGTGATCAATGG, R: GGCAGTGATGGCATGGACTG;
mouse IFN-β-F: GCACTGGGTGGAATGAGACTATTG, R: TTCTGAGGCATCAACTGACAGGTC;
mouse GAPDH-F: ACCACAGTCCATGCCATCA, R: TCCACCACCCTGTTGCTGTA.

### Establishment of mtDNA-depleted ρ^0^ cell lines

HT-29 and PK-15 cells were cultured in DMEM supplemented with 10% FBS, pyruvate (100 μg/mL), uridine (50 μg/mL), ethidium bromide (EB) (500 ng/mL) for 14 days, as previously described [16]. The depletion of mtDNA was confirmed by qPCR using the following primers: human mtDNA-F: CACCCAAGAACAGGGTTTGT, R: TGGCCATGGGTATGTTGTTAA; porcine mtDNA-F: AGCATCCAACTCAAAATACGCAC, R: TCTCCTTCTGTAAGGTCGAACGG; human POLG-F:CTGCCATAAGGTCTGCAGGT, R: CTCCTTTCCGTCAACAGCTC [19]; porcine POLG-F: CCCCAGTCAGTCGCCTTTTTC, R: AATCCCGTATCTCTTTTCCAT. The relative mtDNA amounts were shown as a ratio of mtDNA to nuclear gene POLG.

### Quantification of cGAS-bound mtDNA

Quantification of cGAS-bound mtDNA was performed as described previously [14]. For measurement of mtDNA release *in vivo*, the oropharyngeal tonsils from SVV- or FMDV-infected pigs, and the carcasses from FMDV- or EV-A71-infected mice were collected and minced respectively. Then, the samples were lysed using RIPA buffer for 1 h on ice. For measurement of mtDNA release in cells, the mock-infected or virus-infected cells were collected and lysed, and the lysates were immunoprecipitated with the anti-cGAS antibody for 6 h at 4°C. Total mtDNA isolated from cGAS pulldown was quantified by qPCR analysis, and 20 ng of purified plasmid expressing EGFP gene was added to the extraction liquid. The mix of EGFP and endogenous DNA was used to quantify the presence of specific DNA fragments, as described previously [14]. The EGFP gene was used as an internal control. The qPCR primers used to detect human mtDNA, mouse mtDNA, and human gDNA (ribosomal DNA 18S) have been described previously [14,16]. The qPCR primers used to detect porcine mtDNA and gDNA (GAPDH) were designed in this study. The qPCR primer sequences are shown below. The relative fold changes of DNA molecules of interest were calculated based on the Ct values of EGFP gene amplification.

EGFP-F: ACGGCGACGTAAACGGCCAC, R: GCACGCCGTAGGCTAGGGTG;
human gDNA-F: TAGAGGGACAAGTGGCGTTC, R: CGCTGAGCCAGTCAGTGT;
porcine gDNA-F: GGAGAGGTGTGGTAGCGTTG, R: GGTTTCTGGAAGAGGGGATG;
human mtDNA-F: CACCCAAGAACAGGGTTTGT, R: TGGCCATGGGTATGTTGTTAA;
mouse mtDNA-F: GCCCCAGATATAGCATTCCC, R: GTTCATCCTGTTCCTGCTCC;
porcine mtDNA-F: AGCATCCAACTCAAAATACGCAC, R: TCTCCTTCTGTAAGGTCGAACGG.

### ELISA

Cells supernatant were collected, and the expression of IFN-β protein was detected by human, and porcine IFN-β ELISA kit (Solarbio), according to the manufacturer’s instructions. Cells infected with FMDV, SVV, or EV-A71 were collected, and the level of 2’3’-cGAMP was detected by 2’3’-cGAMP ELISA kit (Cayman Chemical Co., Ann Arbor, MI), according to the manufacturer’s instructions.

### Indirect immunofluorescence microscopy assay

Indirect immunofluorescence microscopy assay (IFA) was performed as mentioned in our previous study [95]. Briefly, cells cultured on Nunc glass bottom dishes (Thermo Fisher Scientific) were infected with SVV, EV-A71, or FMDV. The cells were then fixed with acetone/methanol mixture (1:1) overnight at 4°C and were blocked with 5% normal bovine serum for 6 h at 4°C. After that, the cells were incubated with appropriate primary antibodies overnight at 4°C. The fluorochrome-conjugated secondary antibodies (1:500) were used to react with the primary antibodies in the dark for 6 h at 4°C. Then, the cells were stained with 4’,6-diamidino-2-phenylindole (DAPI) for 10 minutes at room temperature to show the nuclei. The fluorescence was visualized using a Nikon Eclipse 80i fluorescence microscope. The images were captured using NIS Elements F 2.30 software.

### Proteasome and autophagy-lysosome inhibitor assays

Cells cultured in 6-well plates were transfected with empty vector or FLAG-2C expressing plasmids. At 6 h post transfection, the cells were maintained in the fresh medium supplemented with 10% FBS in the presence or absence of proteasomal inhibitor MG132 (20 μM) (Merck & Co., Kenilworth, NJ, USA), autophagy-lysosome inhibitor CQ (100 μM) (Sigma-Aldrich) or 3-MA (1 or 2.5 mM) (Sigma-Aldrich) for 18 h. The collected cells were then analyzed by Western blotting.

Cells cultured in 6-well plates were incubated with SVV or serum-free medium for 1.5 h. Then, cells were maintained in the fresh medium supplemented with 1% FBS in the presence or absence of proteasomal inhibitor MG132 (20 μM), autophagy-lysosome inhibitor CQ (100 μM), and 3-MA (1 or 2.5 mM) for 12 h. After that, cells were harvested for Western blotting analysis.

### Statistical analysis

Statistical analysis was performed using SPSS Statistics for Windows, Version 17.0 (SPSS Inc., Chicago, IL, USA). The unpaired *t* test (two-tailed test analysis) was used in this study. A *p-value <0.05 was considered statistically significant; A **p-value <0.01 was considered statistically highly significant. Data are presented as mean ± SD.

## Supporting information

S1 Fig Picornavirus infection induced mitochondrial damage. (A) PK-15 cells were mock-infected or infected with SVV (MOI 1) for 12 h. The ΔΨm was assessed and compared by evaluation of the state of JC-1 (transformation from JC-1 aggregates to monomers) using a Nikon Eclipse 80i fluorescence microscope. Scale bar, 10 μm. (B) HeLa cells were mock-infected or infected with EV-A71 (MOI 1) for 24 h. PK-15 cells were mock-infected or infected with SVV (MOI 1) or FMDV (MOI 0.5) for 12 h. The mPTP opening was analyzed and compared using a Nikon Eclipse 80i fluorescence microscope. Scale bar, 10 μm.
(TIF)

S2 Fig Picornavirus infection induced mtDNA release. (A) HT-29 cells were mock-infected or infected with EV-A71 (MOI 1) for 24 h. The lysates were immunoprecipitated with an anti-cGAS antibody. The mtDNA and gDNA in the pulldown samples were detected by qPCR. The EGFP DNA was used as an internal control. (B) The mtDNA release in EV-A71-infected HT-29 cells was evaluated by IFA. Cells were double-immunostained for detection of Tom20 (red) and dsDNA (green); cellular nuclei were counterstained with 4’,6-diamidino-2-phenylindole (DAPI) (blue). Scale bar, 10 μm.
(TIF)

S3 Fig Picornavirus infection promoted the level of 2’3’-cGAMP and phosphorylation of STING. PK-15 cells were infected with FMDV or SVV for 6 h (A and B), and Hela cells were infected with EV-A71 for 12 h (C). Poly(dA:dT) was used as a positive control. The level of intracellular 2’3’-cGAMP was detected by 2’3’-cGAMP ELISA kit. The phosphorylation of STING (S366) was determined by Western blotting.
(TIF)

S4 Fig PPID inhibited SVV, FMDV, and EV-A71 replication. The WT and PPID^-/-^ PK-15 cells were infected with SVV (MOI 1) or FMDV (MOI 0.5) for 12 h. The WT and PPID^-/-^ HT-29 cells were infected with EV-A71 (MOI 1) for 24 h. The viral titers of SVV, EV-A71, and FMDV was detected by TCID_50_ assay.
(TIF)

S5 Fig Overexpression of cGAS and STING inhibited picornavirus replication. PK-15 cells transfected with 1.5 μg of empty vector, HA-cGAS and/or HA-STING expressing plasmids were infected with FMDV (MOI 0.5) or SVV (MOI 1) for 12 h, and Hela cells transfected with 1.5 μg of empty vector, HA-cGAS and/or HA-STING expressing plasmids were infected with EV-A71 (MOI 1) for 24 h. The viral titers of FMDV, SVV, and EV-A71 was detected by TCID_50_ assay.
(TIF)

S6 Fig The content of mtDNA in the WT and ρ^0^ cells. The ρ^0^ PK-15 and HT-29 cells were produced as described in “Materials and methods”. The WT and ρ^0^ PK-15 and HT-29 cells were cultured in six-well plates for 24 h, the relative amounts of mtDNA was analyzed by qPCR. Nuclear gene POLG was used as an internal control.
(TIF)

S7 Fig The titers of EV-A71 and FMDV in the WT mice. The three-day-old WT mice were subcutaneously inoculated with FMDV (10^8^ TCID_50_) or EV-A71 (10^8^ TCID_50_) for 0, 1, or 3 d. FMDV and EV-A71 titers from mice were determined by TCID_50_ assay.
(TIF)

S8 Fig The impact of EV-A71 infection on cGAS and STING expression. HT-29 cells and HeLa cells were mock-infected or infected with EV-A71 (MOI 1), respectively. At the indicated time points, the expression of cGAS and STING protein was detected by Western blotting.
(TIF)

S9 Fig The impact of FMDV 2B and 3C on the cGAS-STING signaling pathway. HEK-293T cells were transfected with 1 μg of empty vector or FLAG-2B- and FLAG-3C-expressing plasmids, and 1 μg of empty vector or HA-cGAS plus HA-STING expressing plasmids. At 24 hpt, the IFN-β protein expression level was determined by ELISA.
(TIF)

S10 Fig SVV 2C protein interacted with cGAS. PK-15 cells were mock-infected or infected with SVV (MOI 1) for 12 h. The cells lysates were immunoprecipitated with anti-cGAS (left) or anti-2C (right) antibodies. The antibody-antigen complexes were detected using anti-cGAS and anti-2C antibodies, respectively.
(TIF)

S11 Fig Identification of the functional sites in SVV 2C responsible for cGAS reduction. HEK-293T cells were transfected with 1.5 μg of empty vector, HA-cGAS-, FLAG-2C- or the indicated FLAG-2C-mutants-expressing plasmids. At 24 hpt, the expression of the indicated proteins was determined by Western blotting.
(TIF)

S12 Fig Alignment of the amino acisds sequences of EV-A71, CA16, EMCV, and FMDV 2C. The YS 155–156 sites location is indicated using a red box.
(TIF)
