# Supplementary figures and images for: Innate sensing of picornavirus infection involves cGAS-STING-mediated antiviral responses triggered by mitochondrial DNA release
Source: PLoS Pathog. 2023 Feb 6;19(2):e1011132. doi: 10.1371/journal.ppat.1011132 (PMC9934381; doi:10.1371/journal.ppat.1011132)

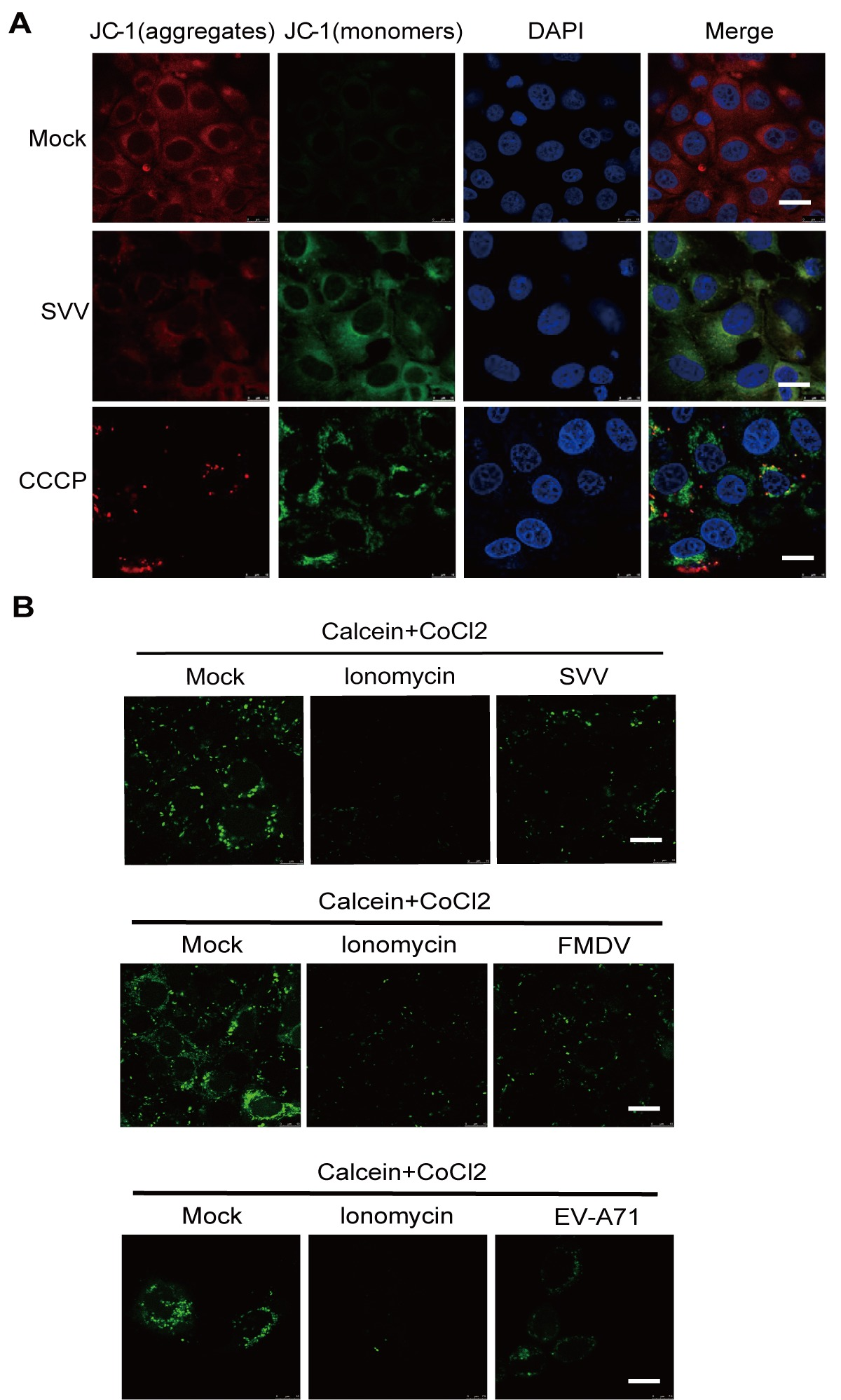

Supplement: S1 Fig — (A) PK-15 cells were mock-infected or infected with SVV (MOI 1) for 12 h. The ΔΨm was assessed and compared by evaluation of the state of JC-1 (transformation from JC-1 aggregates to monomers) using a Nikon Eclipse 80i fluorescence microscope. Scale bar, 10 μm. (B) HeLa cells were mock-infected or infected with EV-A71 (MOI 1) for 24 h. PK-15 cells were mock-infected or infected with SVV (MOI 1) or FMDV (MOI 0.5) for 12 h. The mPTP opening was analyzed and compared using a Nikon Eclipse 80i fluorescence microscope. Scale bar, 10 μm. (TIF) [file ppat.1011132.s001.tif]

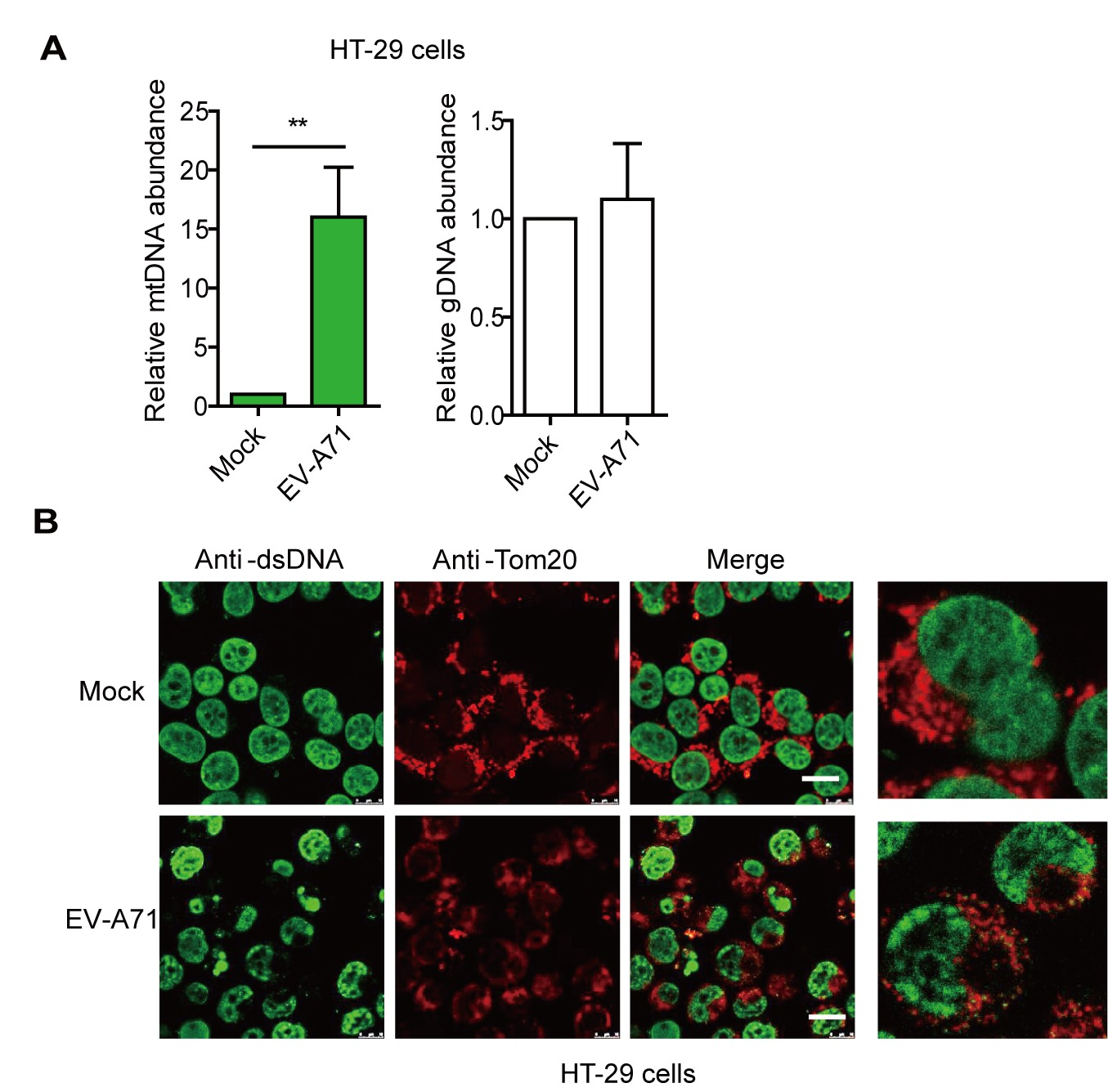

Supplement: S2 Fig — (A) HT-29 cells were mock-infected or infected with EV-A71 (MOI 1) for 24 h. The lysates were immunoprecipitated with an anti-cGAS antibody. The mtDNA and gDNA in the pulldown samples were detected by qPCR. The EGFP DNA was used as an internal control. (B) The mtDNA release in EV-A71-infected HT-29 cells was evaluated by IFA. Cells were double-immunostained for detection of Tom20 (red) and dsDNA (green); cellular nuclei were counterstained with 4’,6-diamidino-2-phenylindole (DAPI) (blue). Scale bar, 10 μm. (TIF) [file ppat.1011132.s002.tif]

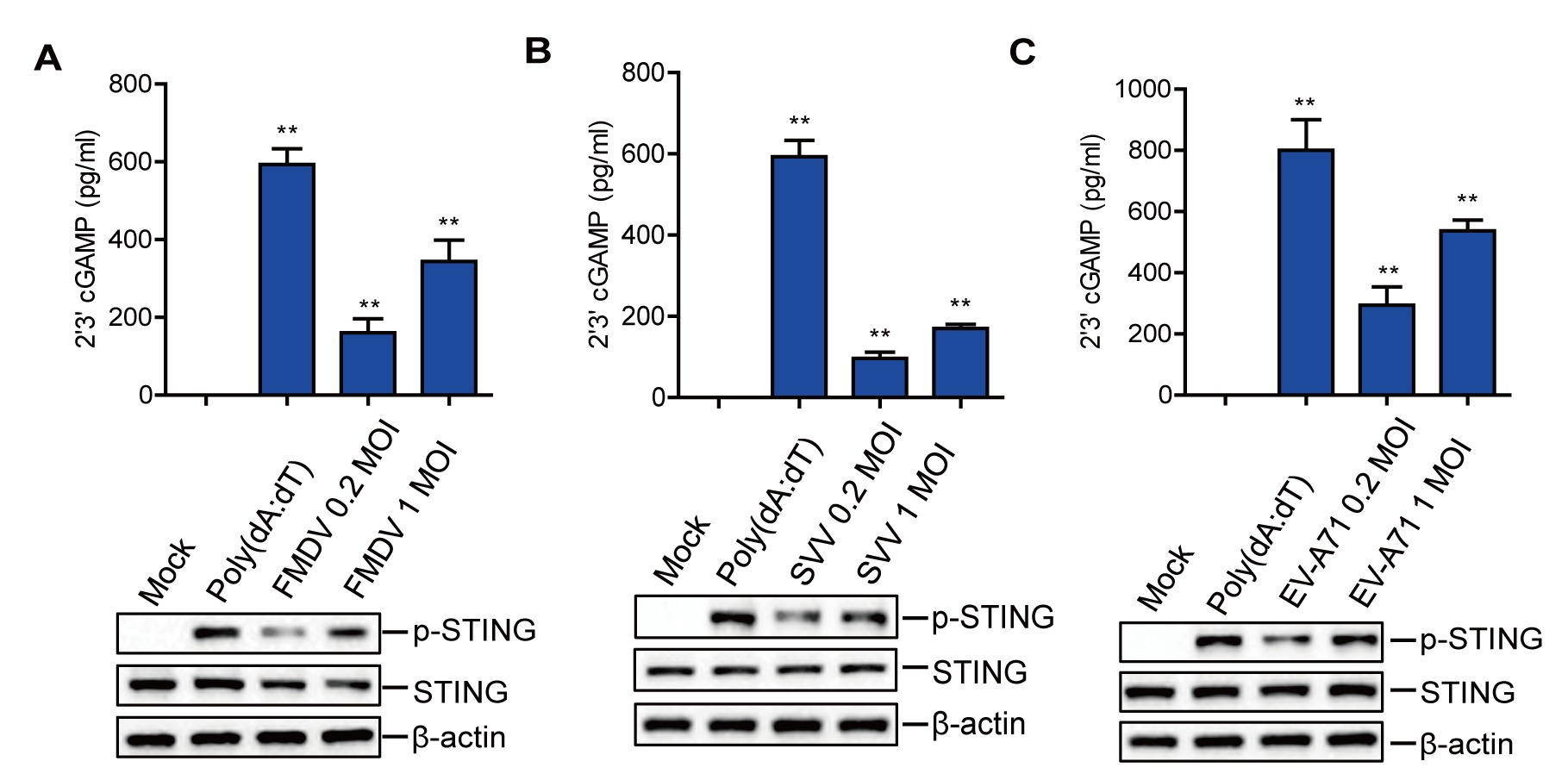

Supplement: S3 Fig — PK-15 cells were infected with FMDV or SVV for 6 h (A and B), and Hela cells were infected with EV-A71 for 12 h (C). Poly(dA:dT) was used as a positive control. The level of intracellular 2’3’-cGAMP was detected by 2’3’-cGAMP ELISA kit. The phosphorylation of STING (S366) was determined by Western blotting. (TIF) [file ppat.1011132.s003.tif]

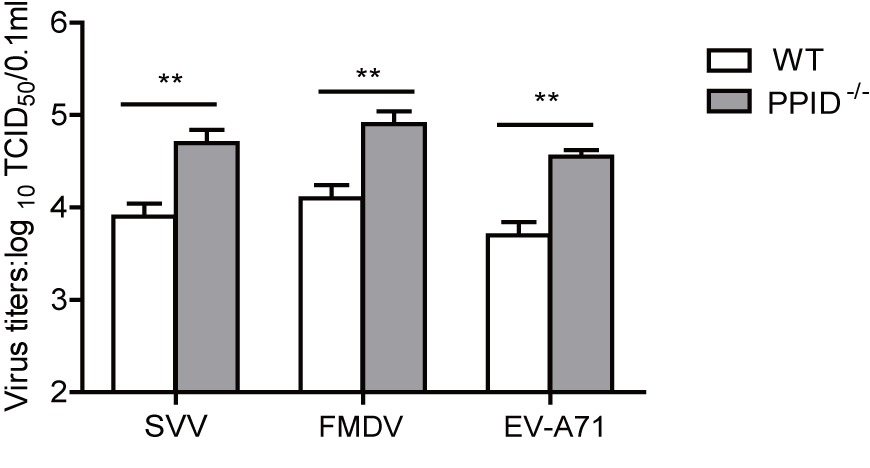

Supplement: S4 Fig — The WT and PPID-/- PK-15 cells were infected with SVV (MOI 1) or FMDV (MOI 0.5) for 12 h. The WT and PPID-/- HT-29 cells were infected with EV-A71 (MOI 1) for 24 h. The viral titers of SVV, EV-A71, and FMDV was detected by TCID50 assay. (TIF) [file ppat.1011132.s004.tif]

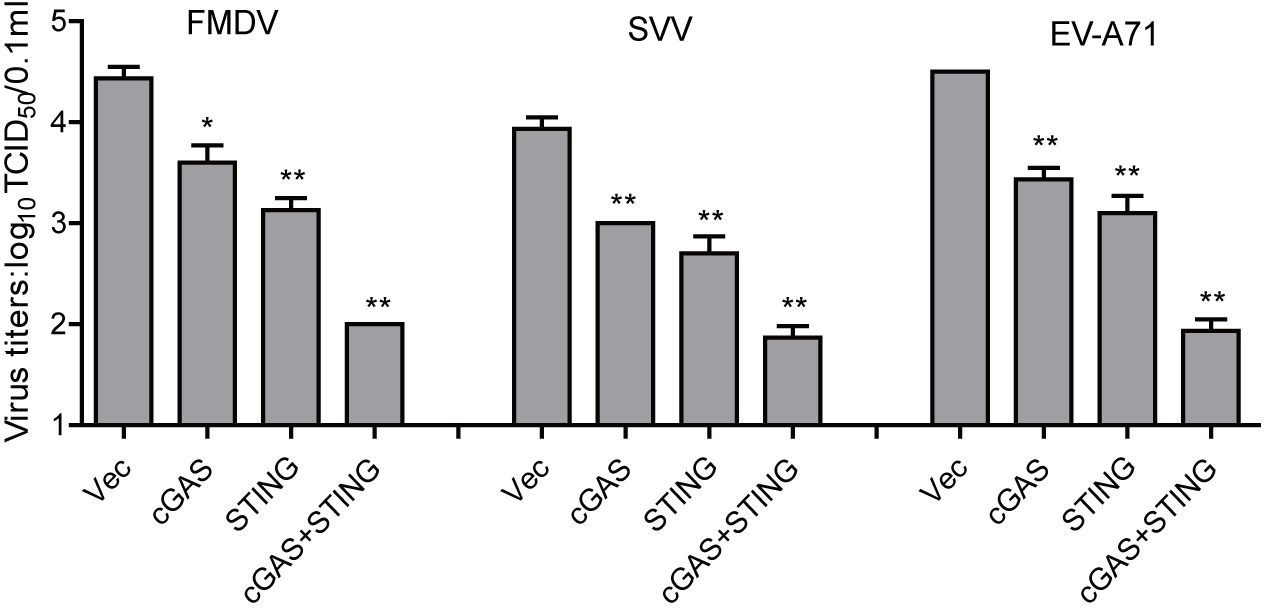

Supplement: S5 Fig — PK-15 cells transfected with 1.5 μg of empty vector, HA-cGAS and/or HA-STING expressing plasmids were infected with FMDV (MOI 0.5) or SVV (MOI 1) for 12 h, and Hela cells transfected with 1.5 μg of empty vector, HA-cGAS and/or HA-STING expressing plasmids were infected with EV-A71 (MOI 1) for 24 h. The viral titers of FMDV, SVV, and EV-A71 was detected by TCID50 assay. (TIF) [file ppat.1011132.s005.tif]

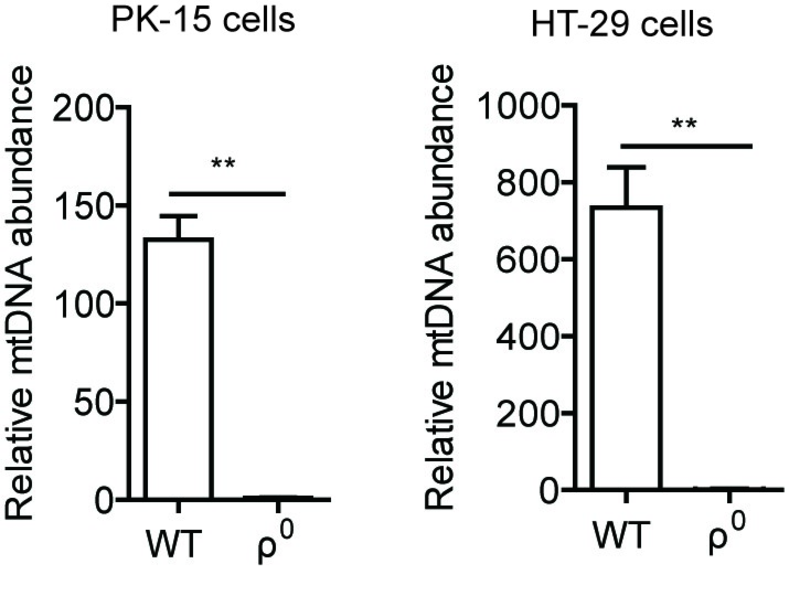

Supplement: S6 Fig — The ρ0 PK-15 and HT-29 cells were produced as described in “Materials and methods”. The WT and ρ0 PK-15 and HT-29 cells were cultured in six-well plates for 24 h, the relative amounts of mtDNA was analyzed by qPCR. Nuclear gene POLG was used as an internal control. (TIF) [file ppat.1011132.s006.tif]

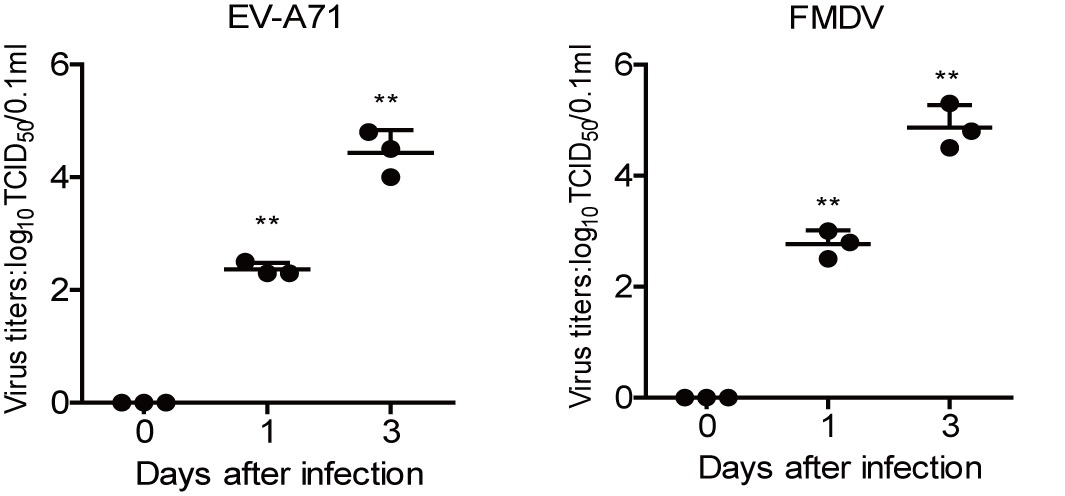

Supplement: S7 Fig — The three-day-old WT mice were subcutaneously inoculated with FMDV (108 TCID50) or EV-A71 (108 TCID50) for 0, 1, or 3 d. FMDV and EV-A71 titers from mice were determined by TCID50 assay. (TIF) [file ppat.1011132.s007.tif]

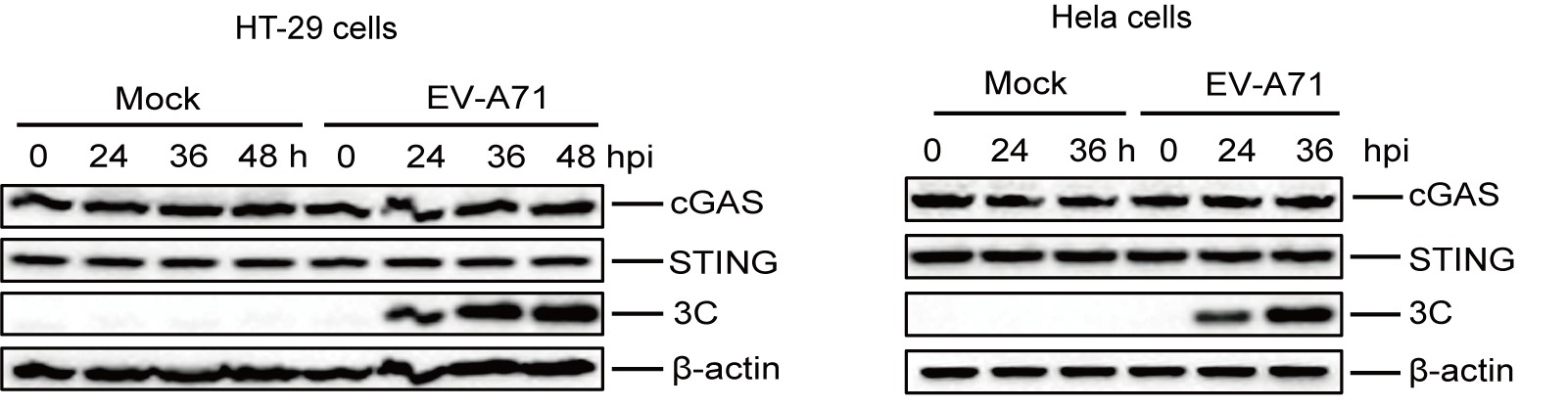

Supplement: S8 Fig — HT-29 cells and HeLa cells were mock-infected or infected with EV-A71 (MOI 1), respectively. At the indicated time points, the expression of cGAS and STING protein was detected by Western blotting. (TIF) [file ppat.1011132.s008.tif]

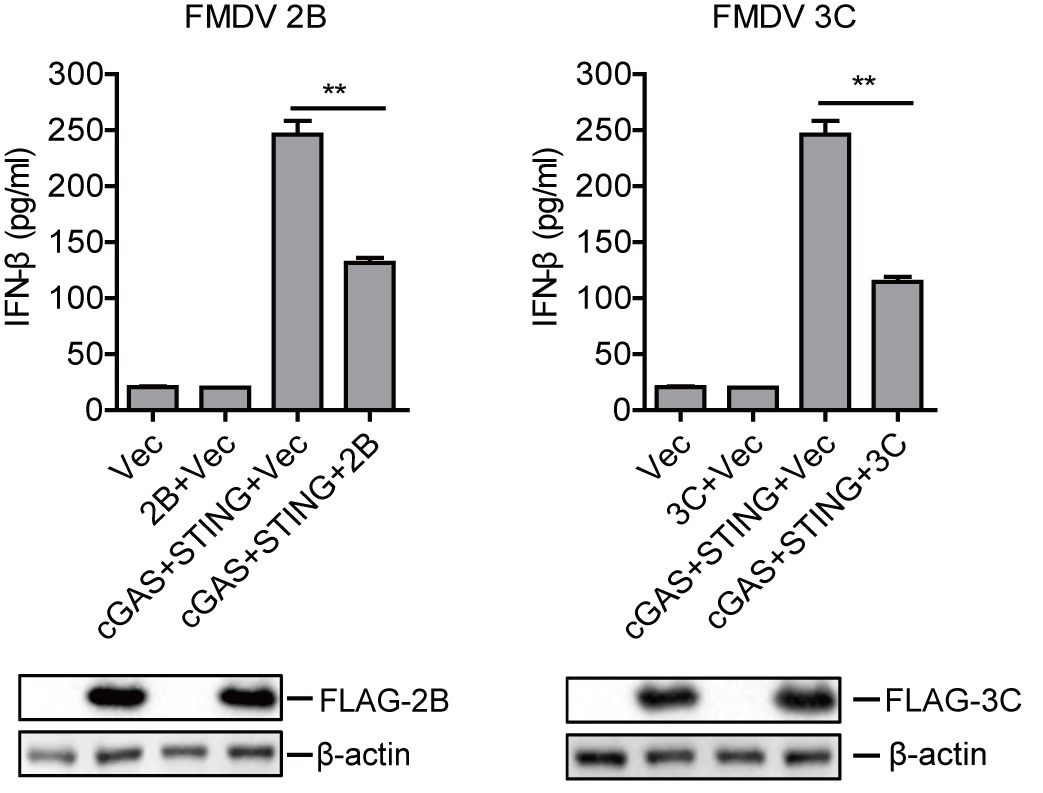

Supplement: S9 Fig — HEK-293T cells were transfected with 1 μg of empty vector or FLAG-2B- and FLAG-3C-expressing plasmids, and 1 μg of empty vector or HA-cGAS plus HA-STING expressing plasmids. At 24 hpt, the IFN-β protein expression level was determined by ELISA. (TIF) [file ppat.1011132.s009.tif]

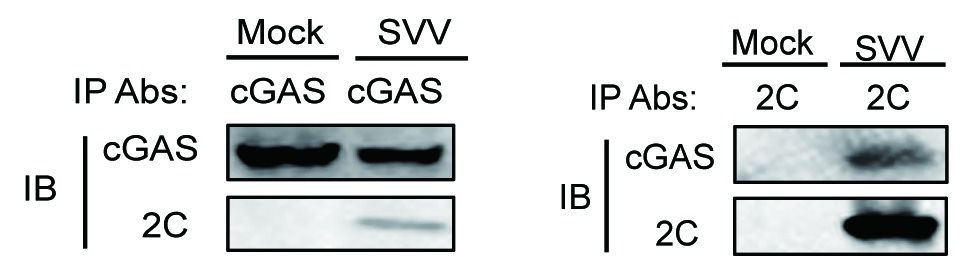

Supplement: S10 Fig — PK-15 cells were mock-infected or infected with SVV (MOI 1) for 12 h. The cells lysates were immunoprecipitated with anti-cGAS (left) or anti-2C (right) antibodies. The antibody-antigen complexes were detected using anti-cGAS and anti-2C antibodies, respectively. (TIF) [file ppat.1011132.s010.tif]

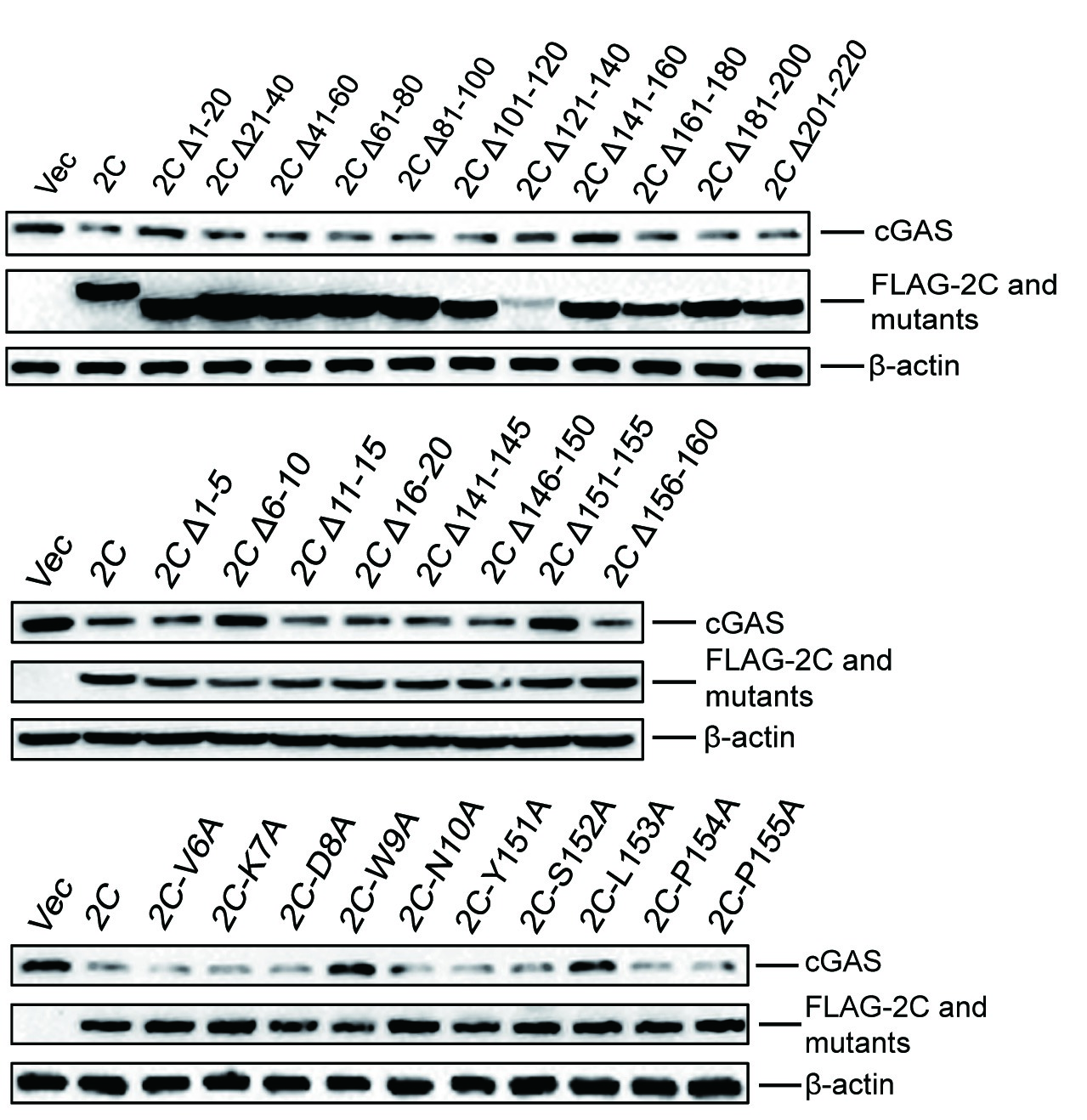

Supplement: S11 Fig — HEK-293T cells were transfected with 1.5 μg of empty vector, HA-cGAS-, FLAG-2C- or the indicated FLAG-2C-mutants-expressing plasmids. At 24 hpt, the expression of the indicated proteins was determined by Western blotting. (TIF) [file ppat.1011132.s011.tif]

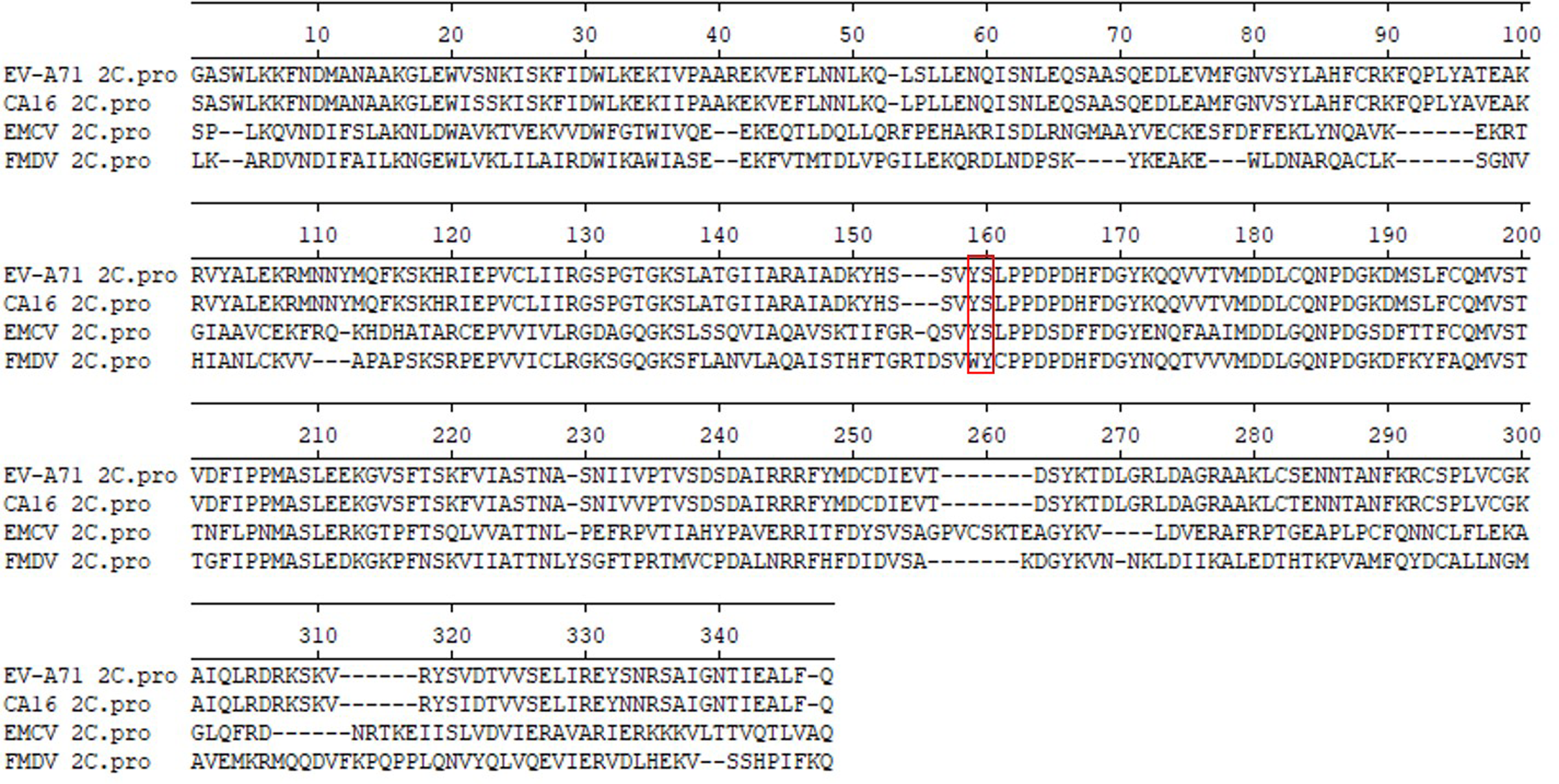

Supplement: S12 Fig — The YS 155–156 sites location is indicated using a red box. (TIF) [file ppat.1011132.s012.tif]
